# Controlled Synthesis of Linear Polyamidoamino Acids

**DOI:** 10.3390/polym11081324

**Published:** 2019-08-08

**Authors:** Federica Ferruti, Jenny Alongi, Amedea Manfredi, Elisabetta Ranucci, Paolo Ferruti

**Affiliations:** Dipartimento di Chimica, Università degli Studi di Milano, via C. Golgi 19, 20133 Milano, Italy

**Keywords:** controlled stepwise polymerization, regularly sequenced polyamidoamino acids, amidoamino acid building blocks, l-arginine, l-alanine

## Abstract

Polyamidoamino acids (PAACs) are synthetic polymers prepared by the polyaddition of bisacrylamides with natural α-amino acids, which in the process maintain both their chirality and their amphoteric nature. This polymerization process is slow, but has the merits of taking place in water and of neither involving protection/de-protection steps nor releasing by-products. However, it leads to polydisperse polymers and, using α-amino acids mixtures, random copolymers. This paper presents a step-by-step polyaddition process leading to homo- and copolymeric PAACs with controlled sequences and controlled molecular weights. It exploits the much different rates of the two Michael addition steps of NH_2_ of α-amino acids with acrylamides, and the low solubility in organic solvents of the α-amino acid addition products. As a proof of principle, the controlled synthesis of the PAAC from l-arginine and *N*,*N*′-methylenebisacrylamide was performed up to a monodisperse product with 11 monomeric units and molecular weight 1840. This synthetic procedure was also tested with l-alanine. All intermediates were isolated and characterized. Noticeably, all of them were α,ω-difunctionalized with either acrylamides or *sec*-amines and were, in fact, building blocks with potential for preparing complex macromolecular architectures. In a first instance, copolymers with controlled sequences of amidoamine- and amidoamino acid units were prepared.

## 1. Introduction

Leaving apart natural and synthetic polypeptides, many examples of bioinspired chiral polymers deriving from natural α-amino acids can be found in the literature [1]. Most of them, prepared by the radical polymerization of *N*-acryloyl- or *N*-methacryloyl α-aminoacids, are in fact polyacrylamides or polymethacrylamide-bearing carboxylated chiral substituents on the nitrogen [2,3,4]. In other polymers, the α-amino acid moieties are attached to polymethacrylate [5], polyacrylate [6], polyacetylene [7,8], polyolefin [9], polyvinyl ether [10], and polyphosphazene [11] backbones. More recently, a new polymer family deriving from natural α-amino acids—polyamidoamino acids (PAACs)—has been described [12,13,14,15]. PAACs are a lateral offshoot of linear polyamidoamines (PAA) [16,17] and, similarly, they are obtained from the polyaddition of natural α-amino acids or their stereoisomers with a bisacrylamide, in particular *N*,*N*′-methylenebisacrylamide. The polymerization takes place in water at pH ≥9.5 in order to deprotonate the amine groups, in the absence of organic solvents or added catalysts. While in the synthesis of most other natural and synthetic polymeric derivatives of α-amino acids, either the amine group, or the carboxyl group, or both lose their acidic or basic properties; in PAACs, these properties, together with chirality, are preserved. Therefore, PAACs are amphoteric. In fact, the polymerization mechanism converts the α-amino acid *prim*-amine group into a *tert*-amine group and does not involve the carboxyl group.

As many other chiral polymers, chiral PAACs and most PAAC/PAA copolymers are capable of assuming stable, pH-dependent conformations in aqueous media [13,14,15]. Owing to the versatility of their chemical structure and their ability to assume chirality-related stable conformations in solution, PAACs constitute a promising and hitherto largely unexplored investigation field for several applications, including biological applications. It may be observed that chirality governs biomaterial-cell interactions [18,19]. In this regard, structure-controlled and narrowly polydisperse polymers would be preferable or even required, but neither have been achieved so far in the synthesis of PAACs and PAAC/PAA copolymers. As for molecular weight control, the polyaddition of amines and α-amino acids with *N*,*N*′-methylenebisacrylamide or other bisacrylamides normally gives products with relatively large polydispersity (PD), normally in the range 1.2–2.67 [12,13,14,15,20,21,22,23,24]. As for structure control, PAACs deriving from α-amino acid mixtures and PAAC/PAA copolymers have the different units randomly distributed along the polymer chain. The aim of this paper is to present a synthetic process capable of filling both gaps.

## 2. Materials and Methods

### 2.1. Materials

Solvents and reagents, unless otherwise indicated, were analytical-grade commercial products and used as received. l-arginine (Ar, ≥98.5%), l-alanine (Al, ≥98%), *N*,*N*′-methylenbisacrylamide (M, 99%), 2,6-di-*tert*-butyl-*p*-cresol (98%), piperazine (Pip, 99%), hydrochloric acid (HCl, 37%), deuterium oxide (D_2_O, 99.9%), and deuterium chloride (DCl, 99%) were purchased from Sigma-Aldrich (Milano, Italy). One molar sodium hydroxide (NaOH) volumetric standard solutions were purchased from Fluka analytics (Milano, Italy).

### 2.2. Characterizations

The ^1^H and ^13^C APT NMR spectra were recorded at pH 4.5 in D_2_O/DCl at 25 °C using a Brüker Avance 500 NMR spectrometer (Bruker, Milano, Italy) operating at 500.130 (^1^H) and 125.758 (^13^C APT) MHz, respectively. ^1^H-^13^C HSQC spectra of MArMArM, ArMArMArMAr, MAlMAlM, and ArMAlMAlMAr were recorded at pH 4.5 in D_2_O/DCl at 25 °C using a Brüker Avance 400 NMR spectrometer (Bruker, Milano, Italy) operating at 400.130 (^1^H) and 100.613 (^13^C APT) MHz, respectively. The weight% purities of all synthesized compounds were determined by ^1^H NMR.

Electrospray ionization mass spectrometry (ESI-MS) analysis was performed using an LCQ Fleet ion trap mass spectrometer (Thermo Fisher Scientific, Monza, Italy) with electrospray source; 0.5 mg mL^−1^ aqueous solutions of the samples were infused into the ion source with a flow rate of 30 μL min^−1^. The instrument was operated both in the positive (ArMAr, MArM, and MArMArM) and negative ion mode (AlMAl, MAlMAlM, and ArMAlMAlMAr). Positive ion mode ESI-MS parameters were set as the following; source voltage: 5.50 kV; source current: 10.00 μA; source temperature: 150.00 °C; sheath gas flow rate: 20.00 (arb); auxiliary gas flow rate: 10.00 (arb); sweep gas flow rate: 5.00 (arb); capillary voltage: −120.00 V; capillary temperature: 275.00 °C; tube lens voltage: 120.00 V. Negative ion mode ESI-MS parameters were set as the following; source voltage: 4.00 kV; source current: 43.00 μA; source temperature: 150.00 °C; sheath gas flow rate: 20.00 (arb); auxiliary gas flow rate: 10.00 (arb); sweep gas flow rate: 5.00 (arb); capillary voltage: −120.00 V; capillary temperature: 275.00 °C; tube lens voltage: −120.00 V.

Molecular weights were determined by Size Exclusion Chromatography (SEC). SEC traces were obtained with Toso-Haas TSK-gel G4000 PW and TSK-gel G3000 PW columns connected in series, using a Waters model 515 HPLC pump equipped with a Knauer autosampler 3800 (Knauer, Bologna, Italy), a Viscotek 270 light-scattering (LALS/RALS) detector (Malvern, Roma, Italy), and a refractive index detector (Waters, Model 2410). The mobile phase was a pH 8.00 ± 0.05 0.1 M Tris buffer solution with 0.2 M sodium chloride. The operational conditions were sample concentration 20 mg mL^−1^, flow rate 1 mL min^−1^, injection volume 20 μL, column dimensions 300 × 7.5 mm^2^, and temperature 25 °C. The instrument optical constants were determined using PEO 19 kDa as a narrow polymer standard. All samples were filtered through a 0.2 μm syringe Whatman filter before measurements. 

### 2.3. Controlled Synthesis of PAACs

*ArMAr.* In a 250 mL one-necked flask, l-arginine (5.426 g, 30.6 mmol) was dissolved in water (60 mL) and the solution maintained at room temperature under stirring. In another flask, an *N*,*N*′-methylenebisacrylamide (2.389 g, 15.3 mmol) and 2,6-di-*tert*-butyl-*p*-cresol (0.059 g, 0.2 mmol) solution in a 1:3 v/v water/methanol mixture (9 mL) was heated to 50 °C until dissolution. The warm solution was added dropwise to the l-arginine solution under stirring. The reaction was allowed to proceed at room temperature for 24 h. The excess methanol was removed under reduced pressure and the remaining solution freeze-dried; yield >98%, purity ≥94%. The ^1^H and ^13^C APT NMR spectra are reported in Figure S1. ESI-MS (m z^−1^) (Figure S12): 503 (M+H)^+^; 525 (M+Na)^+^.

*MArM.* In a 250 mL two-necked flask equipped with a condenser and a silicon rubber stopper, *N*,*N*′-methylenebisacrylamide (26.476 g, 171 mmol) and 2,6-di-*tert*-butyl-*p*-cresol (0.528 g, 2.4 mmol) were dissolved in a 6:1 v/v water/methanol mixture (140 mL), and maintained under stirring at 70 °C. An aqueous l-arginine solution (3.076 g, 17 mmol in 25 mL water) was added dropwise. The resultant solution was continuously stirred at 70 °C for 1 day, stirring was then discontinued, and the reaction mixture maintained 3 days at the same temperature. After this time, the reaction mixture was cooled down to ~0 °C with an ice bath. The major portion of excess *N*,*N*′-methylenebisacrylamide crystallized out and was removed by filtration. The crude product was retrieved from the mother liquors by freeze-drying, then dissolved in a 10:1 v/v methanol/water mixture (25 mL) and poured into acetone (300 mL). MArM was retrieved as a white powder by centrifugation and finally dried under vacuum. Yield 71%, purity ≥98%. Any *N*,*N*′-methylenebisacrylamide still present remained in solution and could be retrieved. The ^1^H and ^13^C APT NMR spectra are reported in Figure S2. ESI-MS (m z^−1^) (Figure S13): 483 (M+H)^+^; 505 (M+Na)^+^.

*MArMArM.* In a two-necked 100 mL flask, *N*,*N*′-methylenebisacrylamide (6.133 g, 39.0 mmol) and 2,6-di-*tert*-butyl-*p*-cresol (0.144 g, 0.6 mmol) were dissolved in a 1:1 v/v water/methanol mixture (40 mL) at 40 °C under stirring. An aqueous ArMAr (2.000 g, 3.9 mmol in 10 mL water) solution was then added dropwise, and the reaction was allowed to proceed at 40 °C for 4 days. The final product was retrieved as described for MArM; yield 63%, purity ≥99%. The ^1^H and ^13^C APT NMR spectra are reported in Figure S3, the ^1^H-^13^C HSQC spectrum is reported in Figure S11a. ESI-MS (m z^−1^) (Figure S14): 811 (M+H)^+^; 833 (M+Na)^+^.

*ArMArMArMAr.* MArMArM (94.3 mg, 0.11 mmol) was dissolved in water (762.3 mg) and l-arginine (44.6 mg, 0.25 mmol) added. The mixture was allowed to react at room temperature for 3 days. The excess l-arginine was removed by ultrafiltration through a membrane with nominal molecular weight cut-off 1000, and the final product retrieved from the retained portion by freeze-drying; yield 57%, purity ≥98%. The ^1^H and ^13^C APT NMR spectra are reported in Figure S4, the ^1^H-^13^C HSQC spectrum is reported in Figure S11b.

*MArMArMArMArM* was prepared as MArMArM by substituting ArMArMArMAr (675.5 mg, 0.58 mmol) for ArMAr; yield 51%, purity ≥98%. The ^1^H and ^13^C APT NMR spectra are reported in Figure S5.

**Figure 1:**
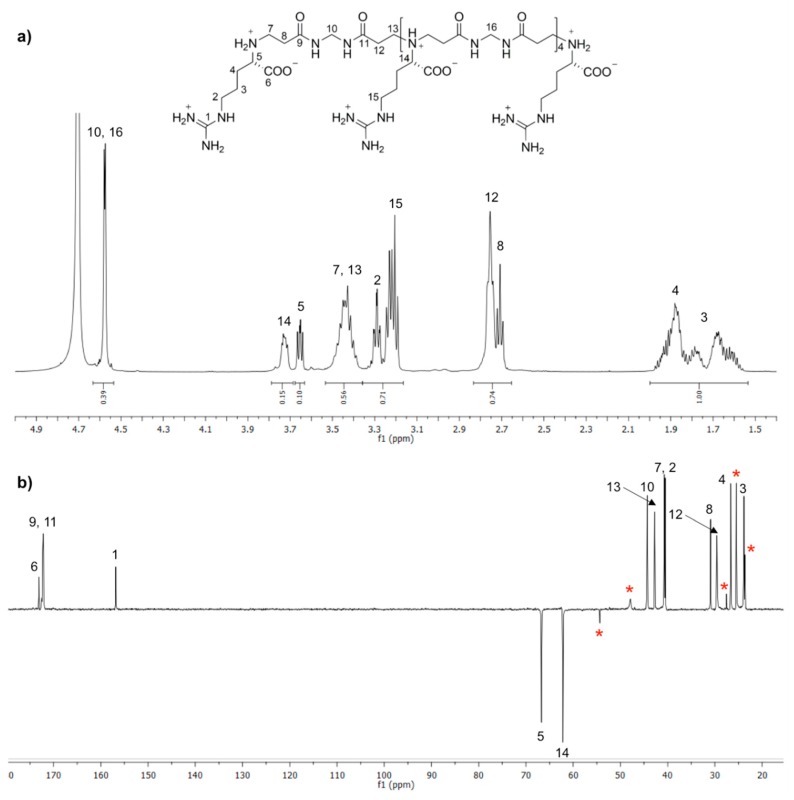
^1^H NMR (**a**) and ^13^C APT NMR (**b**) spectra of ArMArMArMArMArMAr recorded in D_2_O/DCl at pH = 4.5. * Differently protonated species.

*ArMArMArMArMArMAr* was prepared as ArMArMArMAr by substituting MArMArMArM (39.1 mg, 0.03 mmol) for MArMArMArMArM; yield 46%, purity ≥98%. The ^1^H and ^13^C APT NMR spectra are reported in Figure 1.

*AlMAl* was prepared as described for ArMAr by substituting l-alanine (3.006 g, 33.0 mmol) for l-arginine and adding an equimolar amount of NaOH as 1 M aqueous solution; yield >98%, purity ≥98%. The ^1^H and ^13^C APT NMR spectra are reported in Figure S6. ESI-MS (m z^−1^) (Figure S15): 331 (M-H)^−^; 353 (M-2H+Na)^−^.

*MAlMAlM* was prepared as described for MArMArM by substituting AlMAl (1.999 g, 4.9 mmol) for ArMAr. Yield 57%, purity ≥98%. The ^1^H and ^13^C APT NMR spectra are reported in Figure S7, the ^1^H-^13^C HSQC spectrum is reported in Figure S11c. ESI-MS (m z^−1^) (Figure S16): 640 (M-H)^−^; 661 (M-2H+Na)^−^.

*ArMAlMAlMAr* was prepared as ArMArMArMAr by substituting MArMArM for MAlMAlM (0.702 g, 1.09 mmol); yield 43%, purity ≥98%. The ^1^H and ^13^C APT NMR spectra are reported in Figure S8, the ^1^H-^13^C HSQC spectrum is reported in Figure S11d. ESI-MS (m z^−1^) (Figure S17): 988 (M+H)^+^; 1010 (M-2H+Na)^−^.

*[MArM-Pip]_n_*. Piperazine (48.2 mg, 0.55 mmol) was added to an aqueous MArM solution (266.3 mg, 0.55 mmol in 315.0 mg water) and maintained under stirring until complete dissolution. The reaction was maintained at 25 °C for 5 days with occasional stirring. After this time, the reaction mixture was acidified to pH 3.5 with 6 M HCl and ultrafiltered through a membrane with nominal molecular weight cut-off 3000. The product was then retrieved from the retained portion by freeze-drying; yield 42%. *M_w_* = 7250; *M_w_/M_n_* = 1.11. The ^1^H and ^13^C APT NMR spectra are reported in Figure S9. The SEC traces are reported in Figure S18.

*[MArMArM-Pip]_n_* was prepared as [MArM-Pip]_n_ by substituting MArMArM (0.509 g, 0.55 mmol) for MArM. The reaction proceeded for 17 days; yield 37%. *M_w_* = 5270; *M_w_/M_n_* = 1.09. The ^1^H and ^13^C APT NMR spectra are reported in Figure S10. The SEC traces are reported in Figure S19.

## 3. Results and Discussion

### 3.1. Rationale of the Synthetic Process

The synthetic process developed for the controlled synthesis of PAAC exploits the wide difference in reactivity normally existing between the N-H groups of the α-amino acids in the Michael addition with activated double bonds, as reported for instance for the addition reaction of l-valine with hydroxymethyl-vinyl ketone, which prevailingly yields the monoadduct [25]. Besides that, the process exploits the well-known low solubility in organic solvents of α-amino acids and their addition products. Both factors made it possible to prepare oligomeric building blocks and to proceed further step-by-step without requiring either solid or soluble supports, or tedious protection/de-protection procedures. Furthermore, the process is based on the Michael type addition reaction and therefore does not release by-products to be eliminated at the end of each step.

### 3.2. Stepwise Controlled Synthesis of PAAC Homo- and Copolymers

The controlled synthesis of the PAAC called l-ARGO7 [12,13], and of its copolymers with l-alanine as a model for other amino acids, has provided the proof of principle of the new process. l-ARGO7 and its d- and d, l-stereoisomers with broad polydispersity had been previously prepared by polyaddition of l-arginine (henceforth called Ar) with *N*,*N*′-methylenebisacrylamide (henceforth called M). l-ARGO7, which is the first PAAC ever reported in the literature, is nontoxic and easily internalized in cells [12]. The first step of the controlled synthesis of l-ARGO7 was preparing the ArMAr trimer or the MArM trimer. 

**Scheme 1:**
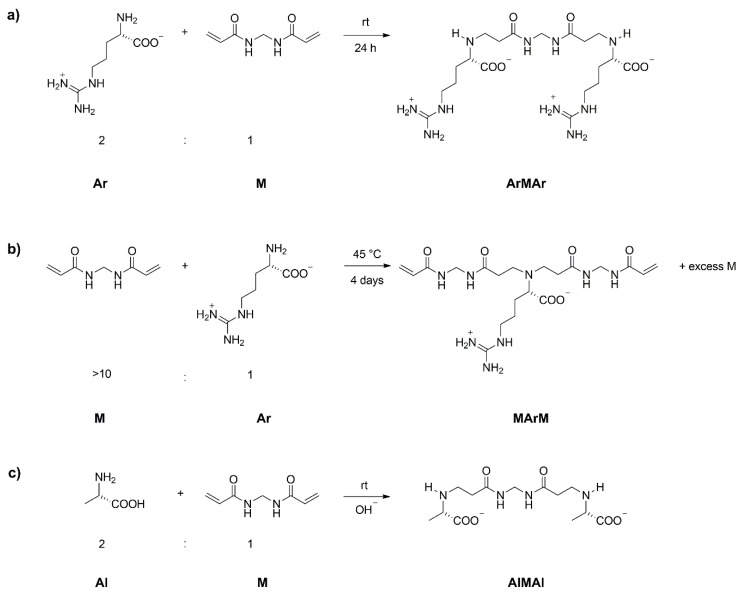
Synthesis of l-arginine- and l-alanine-based trimers.

In the first instance (Scheme 1a), an M/Ar mixture in a 1:2 molar ratio at 20–25 °C in water, straightforwardly yielded after 24 h ~99% pure ArMAr that was retrieved by freeze-drying. The poor reactivity of the NH_2_ of l-arginine in the second Michael addition step prevented formation of higher molecular weight oligomers. It should be noticed that the amine group of l-arginine is not protonated, since the guanidine group neutralizes the carboxyl group. 

In the second instance (Scheme 1b), treating 1 mole Ar with ≥10 moles M and forcing the reaction by heating at 40–45 °C for 4 days, a mixture of MArM and excess M was obtained after freeze-drying. The excess M was removed by dissolving the crude product in wet methanol and reprecipitating with acetone. The pure trimer was filtered off and the excess M recovered by evaporating the solvent.

**Scheme 2:**
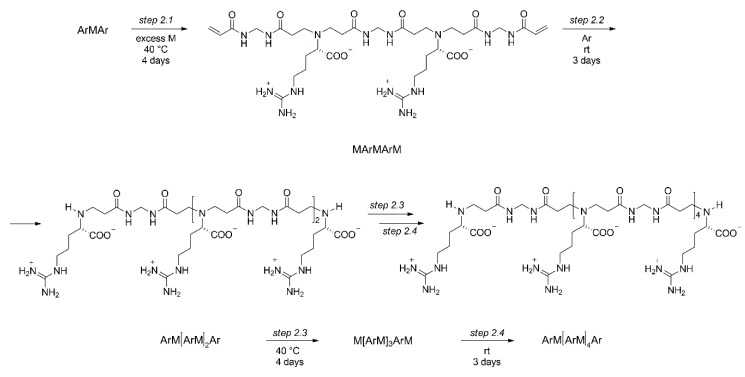
Synthesis of l-arginine-based building blocks.

The above trimers constituted the starting building blocks from which the controlled synthesis of PAACs was performed. In particular, ArMAr was reacted with excess M under the same forcing conditions adopted as described in Scheme 1b, giving the MArMArM pentamer (Step 2.1, Scheme 2), which in turn was reacted with 2 mol Ar as described in Scheme 1a, giving the ArMArMArMAr heptamer (Step 2.2, Scheme 2), which in turn was reacted with excess M as in Step 2.1, and so on. The process, in principle, could have been continued indefinitely, but in the present work was deliberately stopped after obtaining the ArMArMArMArMArMAr undecamer (Step 2.4, Scheme 2) with molecular weight 1814, whose structure was confirmed by ^1^H and ^13^C APT NMR (Figure 1), as all the intermediates above described (Figures S1–S5 of the Supplementary Materials). In addition, for MArMArM and ArMArMArMAr ^1^H-^13^C HSQC spectra are reported in Figure S11a,b. The trimers ArMAr and MArM trimers as well as the MArMArM pentamer were characterized also by ESI-MS spectrometry (Figures S12–S14).

It may be observed that as long as the step-by-step process proceeds, the isolation and purification of the resultant intermediates becomes easier, since the difference in molecular weight between the substrate and excess reagents becomes larger, any molar excess reagent becomes less relevant on a weight/weight basis and any size-based purification method more effective. In fact, the purity of the final product increases from the pentamer upwards.

Obviously, the same procedure could also have been applied starting from MArM by reacting it first with 2 mol Ar, then with excess M, and so on. 

**Scheme 3:**
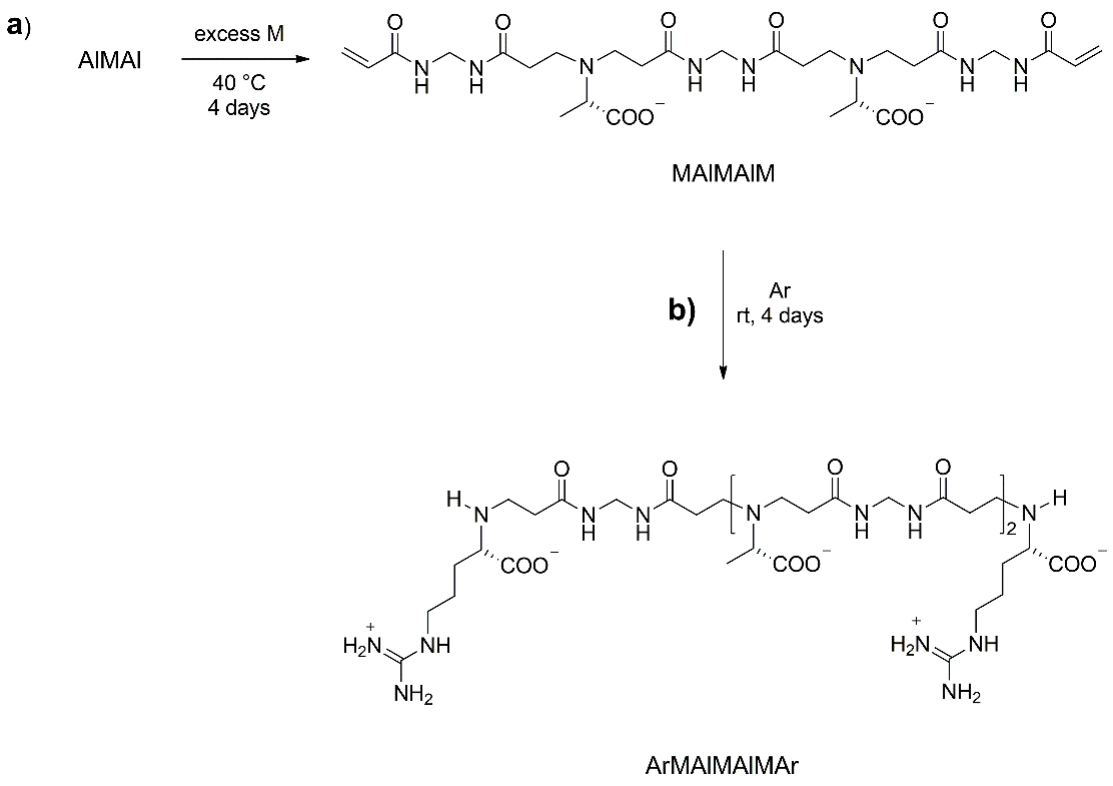
Synthesis of l-arginine/l-alanine-based copolymeric building blocks.

It is evident that the scope of the process is not limited to the controlled synthesis of l-ARGO7, but can also be applied to the synthesis of other PAACs, with the only difference, henceforth implicit, that in the case of neutral amino acids it is necessary to deprotonate the amine group by adding an equimolecular quantity of a base to the reaction recipe. Moreover, a different amino acid unit can be inserted at every step. For example, by substituting l-alanine (Al) for Ar, the l-ARGO7 preparation procedures were used to prepare the AlMAl trimer (Scheme 1c), which in turn was reacted with excess M obtaining the MAlMAlM pentamer (Scheme 3a) that was further reacted with Ar, obtaining the copolymeric ArMAlMAlMAr heptamer (Scheme 3b). Therefore, the process allows preparation not only PAAC samples with controlled molecular weight, but also PAAC copolymers with controlled molecular weight and controlled sequences of amino acid units. Also these intermediates were isolated and characterized by ^1^H, ^13^C APT and ^1^H-^13^C HSQC NMR spectrometry (Figures S7, S8, S11c, and S11d of Supplementary Materials) and by ESI-MS spectrometry (Figures S15–S17). 

### 3.3. Use of Intermediates as Building Blocks for Preparing PAAC-PAA Copolymers

**Scheme 4:**
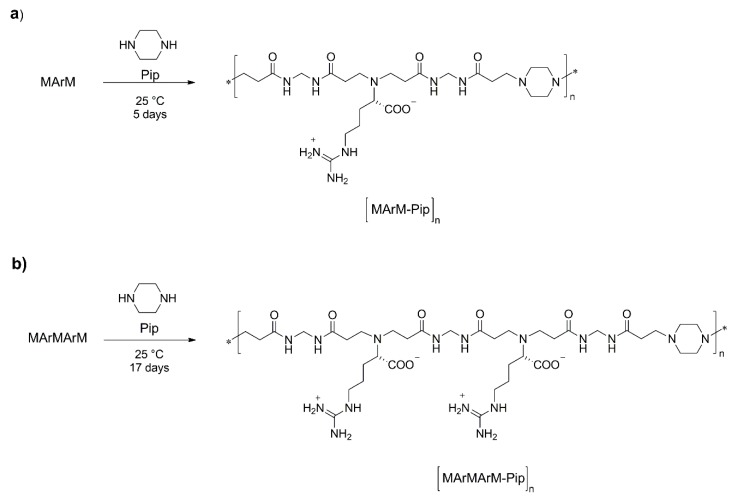
Synthesis of l-arginine/piperazine based amidoamino acid/amidoamine copolymers.

All α, ω-M-terminated intermediates from MArM upwards are, in fact, bisacrylamides. As such, they react with *prim*-monoamines and *sec*-bisamines to give linear copolymeric PAACs or PAAC/PAA copolymers with controlled amido-α-aminoacid/amidoamine sequences. As a proof of principle, the polyadditions of MArM and MArMArM with equimolecular amounts of piperazine were performed in water at room temperature, obtaining regularly sequenced copolymers with [MArM-Pip]_n_ (Scheme 4a) and [MArMArM-Pip]_n_ (Scheme 4b) structures. Both copolymers were isolated by freeze-drying after a single ultrafiltration step through a membrane with a molecular weight cut-off of 3000. 

[MArM-Pip]_n_ and [MArMArM-Pip]_n_ had *M_n_* 6500 (PD 1.11) and 4800 (PD 1.09) (SEC traces reported in Figures S18 and S19), respectively. ^1^H NMR confirmed these determinations. The number of repeat units in both copolymers were calculated from the ratio of the C-H integrals of the arginine stereocenters present in the internal repeating units over those of the arginine units adjacent to the piperazine-functionalized terminals (Figure S9a for [MArM-Pip]_n_ and Figure S10a for [MArMArM-Pip]_n_). The absence of residual acrylamide double bonds allowed to hypothesize that both terminals were equally functionalized with piperazine moieties. The obtained values, 6900 ([MArM-Pip]_n_) and 4500 ([MArMArM-Pip]_n_) were in good agreement with the SEC results. Therefore, besides having a controlled structure, these copolymers had narrow molecular weight distributions, approaching monodispersity. A possible explanation is that in these alternating PAAC-PAA copolymers the oligomeric PAAC blocks had a much higher molecular weight (482 and 810, respectively) than piperazine (86), and therefore on a weight-to-weight basis they constituted the by far prevailing portion (~85% and 90.5%, respectively). The polymerization degrees and number of molecular species of [MArM-Pip]_n_ and [MArMArM-Pip]_n_ present in samples of molecular weight of a few thousands was necessarily modest compared with that of a usual PAA or PAAC of similar molecular weight. During ultrafiltration, to be retained by- or to pass through the membrane was probably a matter of one or two building block units. For all these reasons, the fractionation proceeded through sharp molecular weight “jumps” and significantly narrowed polydispersity.

## 4. Conclusions

The results reported in this paper demonstrate that the controlled synthesis of linear PAACs both in terms of unit sequences and molecular weight is feasible. The step-by-step process devised is slow but has the advantages of simplicity and absence of secondary by-products. Moreover, all intermediates obtained in each step are alternatively α, ω-terminated by either acrylamide double bonds or *sec*-amine groups. Therefore, they actually constitute macromonomers that can be used as building blocks to prepare PAAC–PAA copolymers in which the different units are regularly sequenced along the polymer chain. Both types of copolymers after a single ultrafiltration step are almost monodisperse, therefore, the use of intermediate oligomers as building blocks may constitute a facile short cut to PAAC-PAA copolymers with controlled sequences and with narrow polydispersity. As a final conclusion, the synthetic process devised has a wide potential for a new category of synthetic polymers with controlled sequence of monomer units and controlled molecular weight, prepared using natural α-amino acids or their stereoisomers as monomers.

## Supplementary Materials

The following are available online at https://www.mdpi.com/2073-4360/11/8/1324/s1. Figure S1: ^1^H NMR (a) and ^13^C APT NMR; (b) spectra of ArMAr recorded in D_2_O/DCl at pH = 4.5. Figure S2: ^1^H NMR (a) and ^13^C APT NMR; (b) spectra of MArM recorded in D_2_O/DCl at pH = 4.5. Figure S3: ^1^H-NMR (a) and ^13^C APT NMR; (b) spectra of MArMArM recorded in D_2_O/DCl at pH = 4.5. Figure S4: ^1^H NMR (a) and ^13^C APT NMR; (b) spectra of ArMArMArMAr recorded in D_2_O/DCl at pH = 4.5. Figure S5: ^1^H NMR (a) and ^13^C APT NMR; (b) spectra of MArMArMArMArM recorded in D_2_O/DCl at pH = 4.5. Figure S6: ^1^H NMR (a) and ^13^C APT NMR; (b) spectra of AlMAl recorded in D_2_O/DCl at pH = 4.5. Figure S7: ^1^H NMR (a) and ^13^C APT NMR; (b) spectra of MAlMAlM recorded in D_2_O/DCl at pH = 4.5. Figure S8: ^1^H NMR (a) and ^13^C APT NMR; (b) spectra of ArMAlMAlMAr recorded in D_2_O/DCl at pH = 4.5. Figure S9: ^1^H NMR (a) and ^13^C APT NMR; (b) spectra of [MArM-Pip]_n_ recorded in D_2_O/DCl at pH = 4.5. Figure S10: ^1^H NMR (a) and ^13^C APT NMR; (b) spectra of [MArMArM-Pip]_n_ recorded in D_2_O/DCl at pH = 4.5. Figure S11: ^1^H-^13^C HSQC spectra of MArMArM (a); ArMArMArMAr (b); MAlMAlM (c); ArMAlMAlMAr (d) recorded in D_2_O/DCl at pH N 4.5. Figure S12: Positive ionization mode ESI-MS spectrum of ArMAr. Figure S13: Positive ionization mode ESI-MS spectrum of MArM. Figure S14: Positive ionization mode ESI-MS spectrum of MArMArM. Figure S15: Negative ionization mode ESI-MS spectrum of AlMAl. Figure S16: Negative ionization mode ESI-MS spectrum of MAlMAlM. Figure S17: Negative ionization mode ESI-MS spectrum of ArMAlMAlMAr. Figure S18: Snapshot of the OminiSEC 4.2 software output showing the results of SEC analysis of the [MArM-Pip]_n_ copolymer carried out at pH 8.00 ± 0.05 0.1 M Tris buffer solution with 0.2 M sodium chloride. The operational conditions were: sample concentration 20 mg mL^−1^; flow rate 1 mL min^−1^; injection volume 20 μL; column dimensions 300 × 7.5 mm^2^, temperature 25 °C. Figure S19: Snapshot of the OminiSEC 4.2 software output showing the results of SEC analysis of the [MArMArM-Pip]_n_ copolymer carried out at pH 8.00 ± 0.05 0.1 M Tris buffer solution with 0.2 M sodium chloride. The operational conditions were: sample concentration 20 mg mL^−1^; flow rate 1 mL min^−1^; injection volume 20 μL; column dimensions 300 × 7.5 mm^2^, temperature 25 °C. Green line: light-scattering trace, red line: refractive index trace.
