# Supplementary material for: Controlled Synthesis of Linear Polyamidoamino Acids
Source: Polymers (Basel). 2019 Aug 8;11(8):1324. doi: 10.3390/polym11081324 (PMC6722684; doi:10.3390/polym11081324)
Supplement: Supplementary file 1 [file polymers-11-01324-s001.pdf]

Supplementary Materials

Controlled Synthesis of Linear Polyamidoamino Acids

Federica Ferruti , Jenny Alongi, Amedea Manfredi, Elisabetta Ranucci* and Paolo Ferruti*

Dipartimento di Chimica, Università degli Studi di Milano, via C. Golgi 19, 20133 Milano, Italy; federicamariacamillaferruti@studenti.unimi.it (F.F.); jenny.alongi@unimi.it (J.A.); amedea.manfredi@unimi.it (A.M.)

* Correspondence: elisabetta.ranucci@unimi.it (E.R.); paolo.ferruti@unimi.it (P.F.); Tel.: +39–02-50314132 (E.R.), +39–02-5031-4128 (P.F.)

Figures S1-S10. ^{1}H NMR (a) and ^{13}C APT NMR (b) spectra.

Figure S11. ^{1}H-^{13}C HSQC spectra.

Figures S12-S17. ESI-MS spectra.

Figures S18-S19. SEC traces.

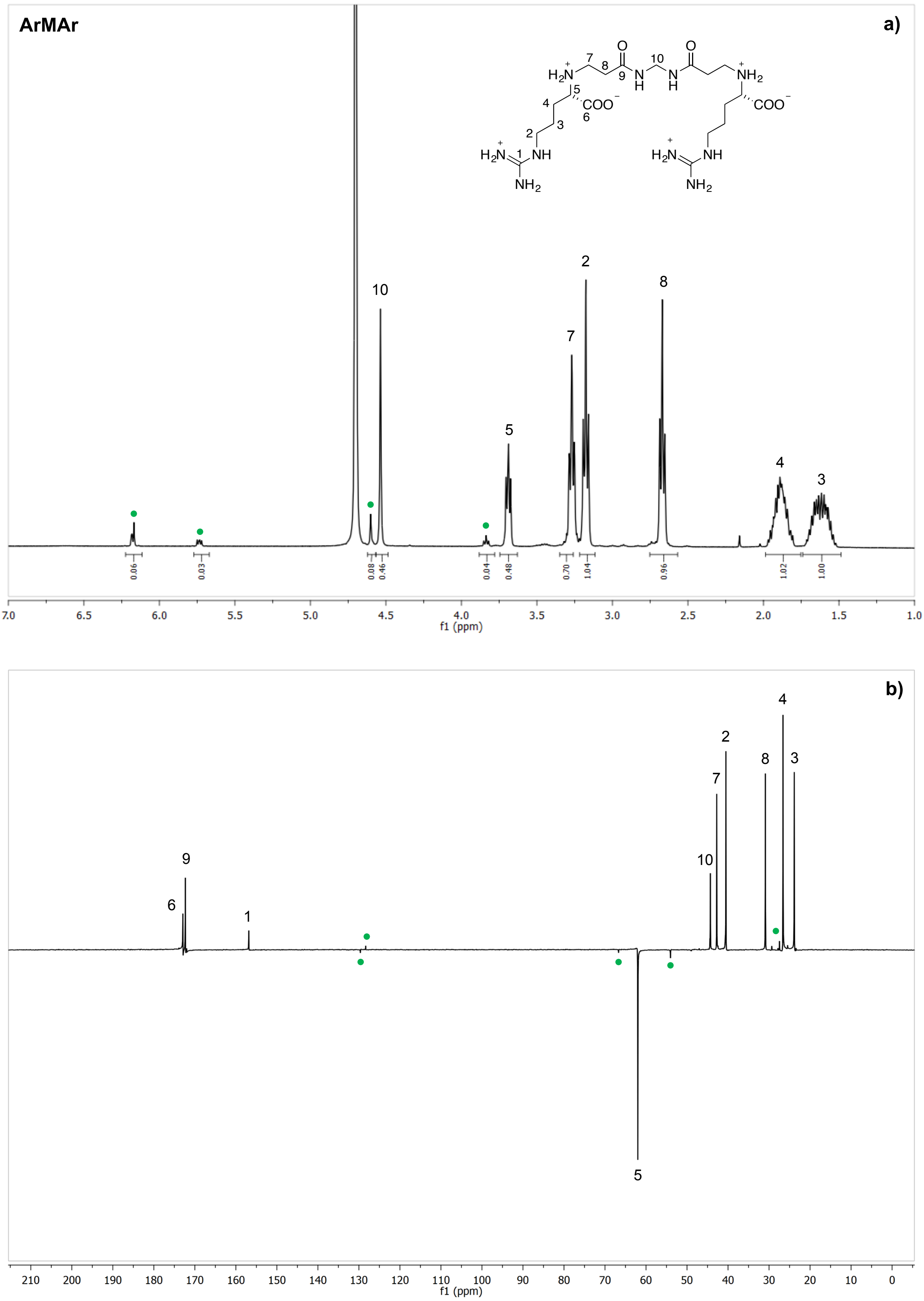

Figure S1. ^{1}H NMR (a) and ^{13}C APT NMR (b) spectra of ArMAr recorded in D_2O/DCl at pH = 4.5.

• ArM dimer impurity

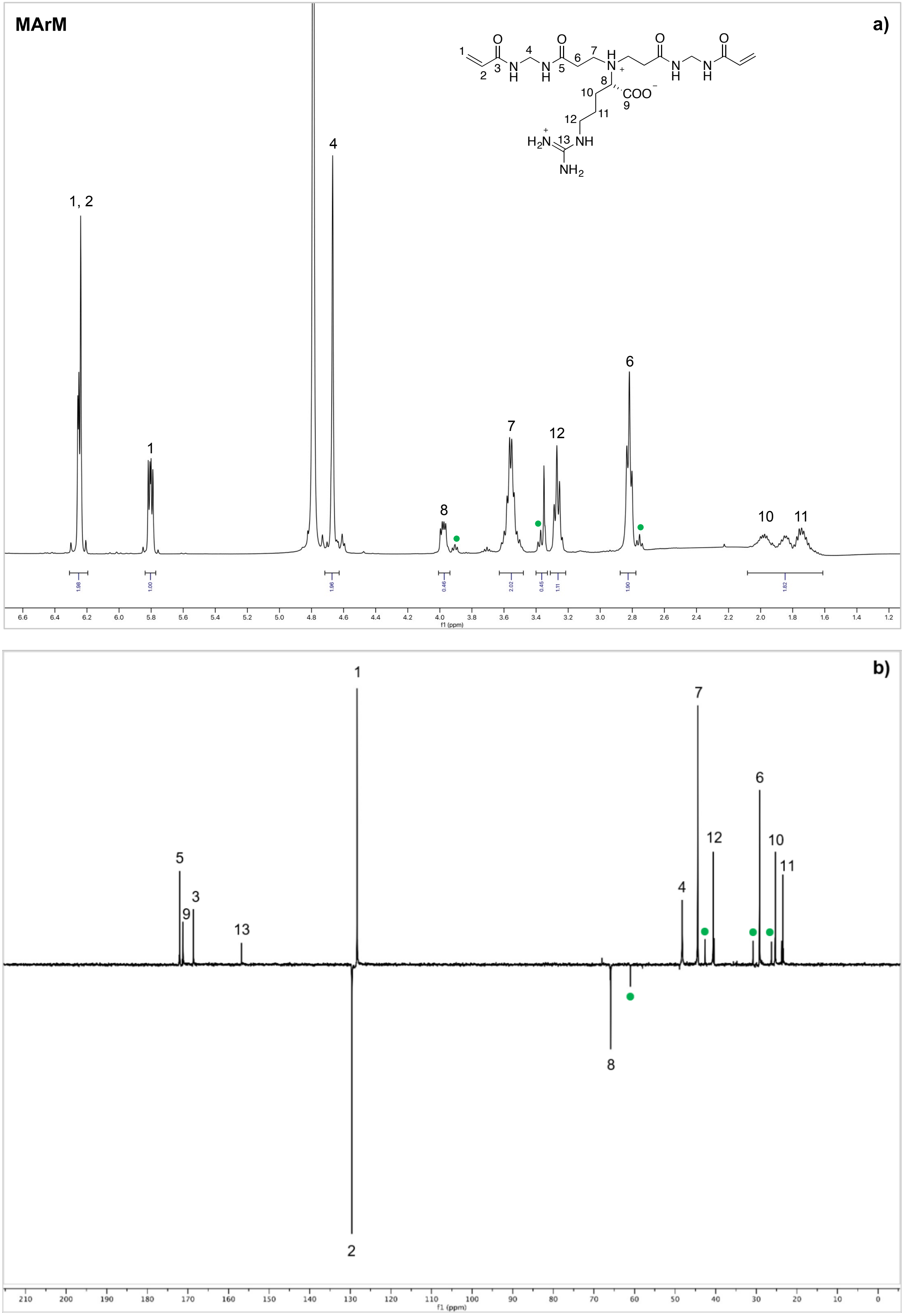

Figure S2. ^{1}H NMR (a) and ^{13}C APT NMR (b) spectra of MArM recorded in D_2O/DCl at pH = 4.5.

• MArMArM pentamer impurity

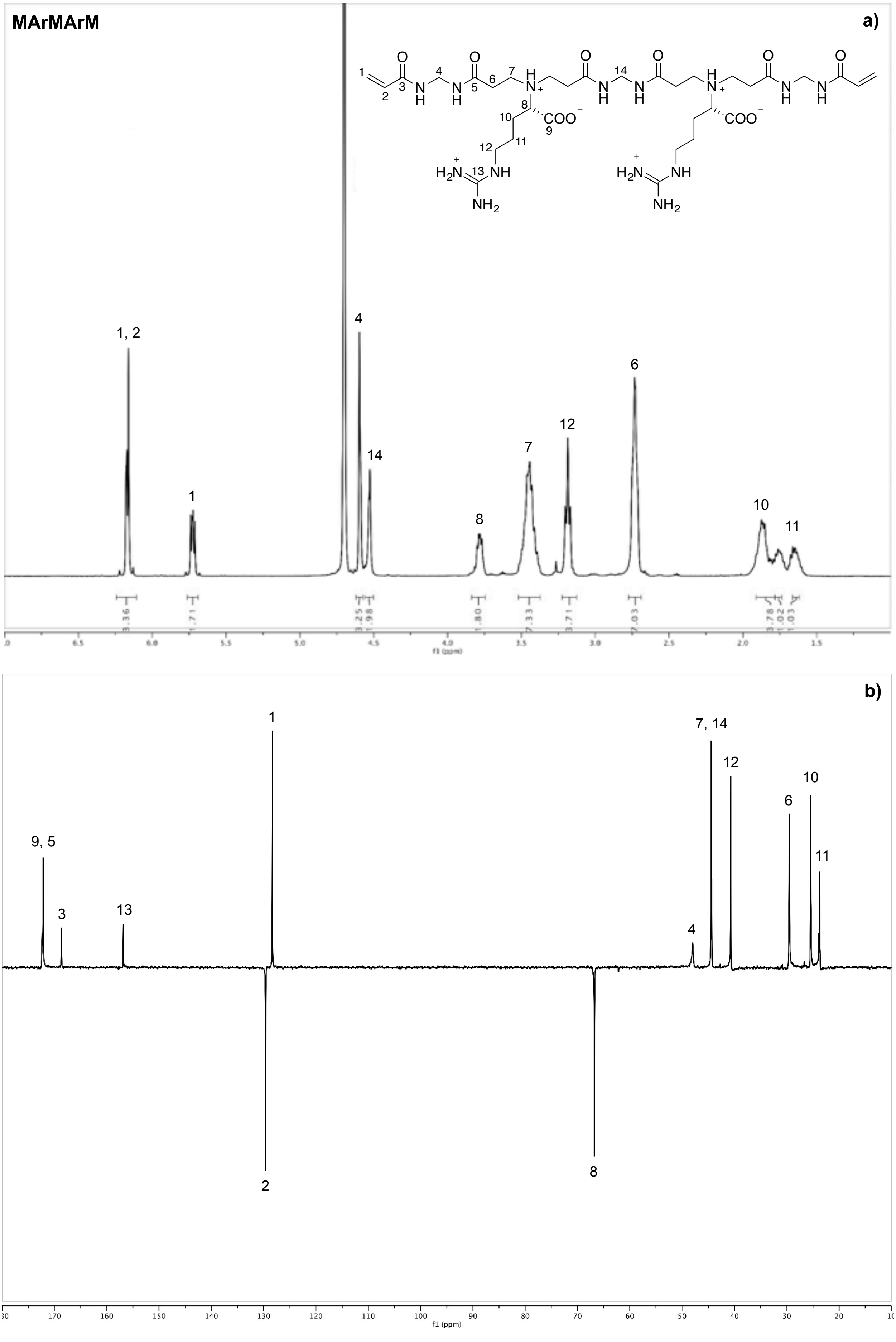

Figure S3. ^{1}H-NMR (a) and ^{13}C APT NMR (b) spectra of MArMArM recorded in D_2O/DCl at pH = 4.5.

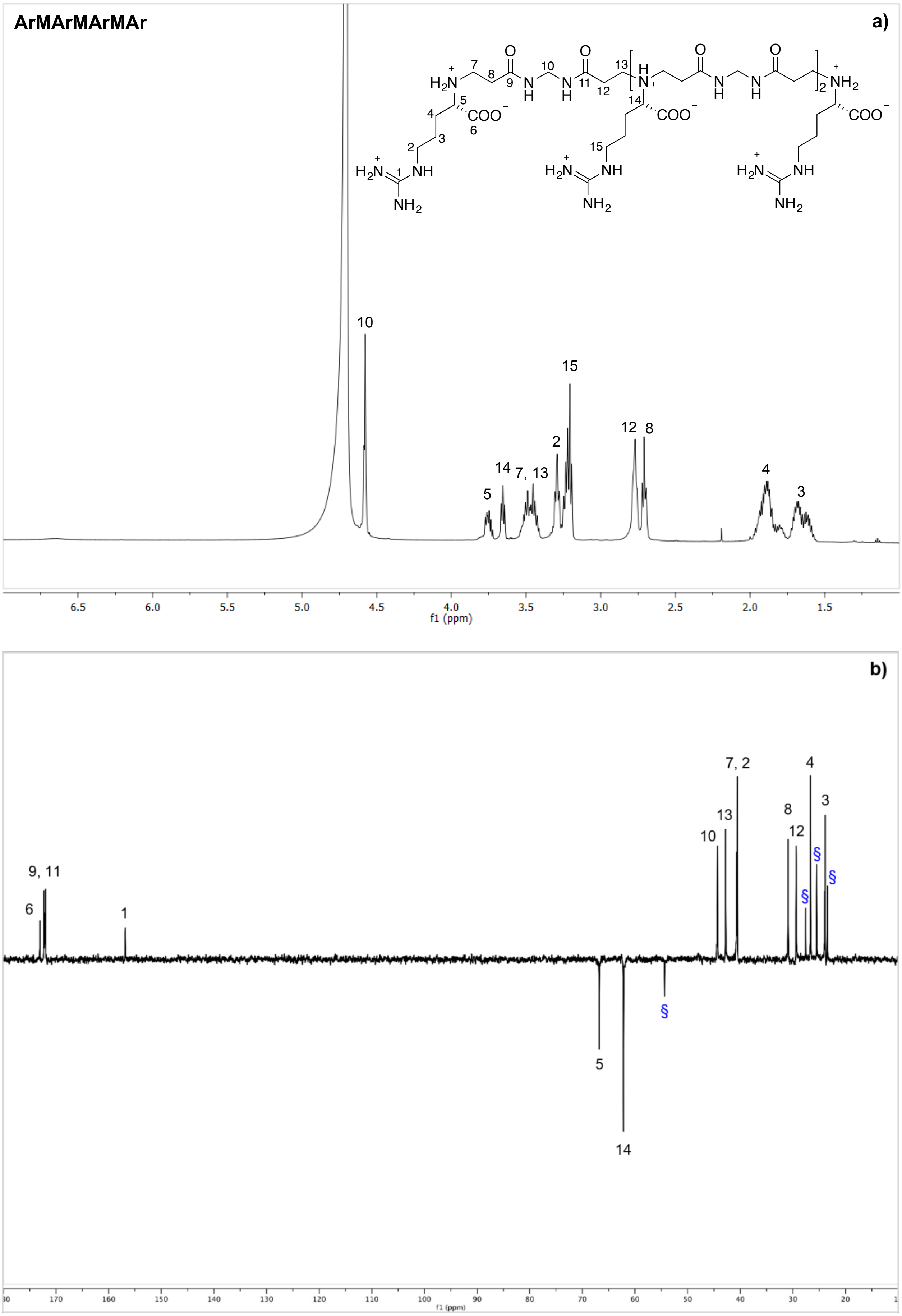

Figure S4. ^{1}H NMR (a) and ^{13}C APT NMR (b) spectra of ArMArMArMAr recorded in D_2O/DCl at pH = 4.5.

§ *L*-Arginine impurity

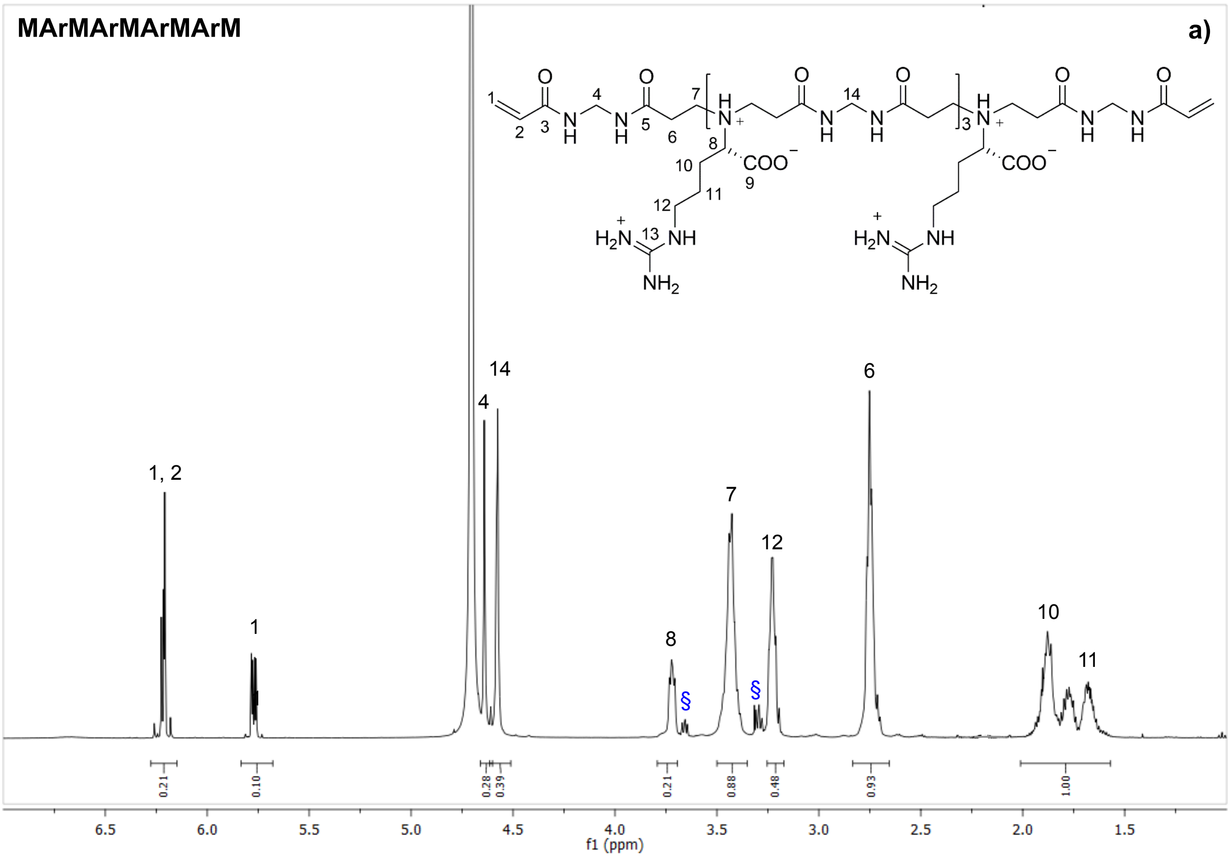

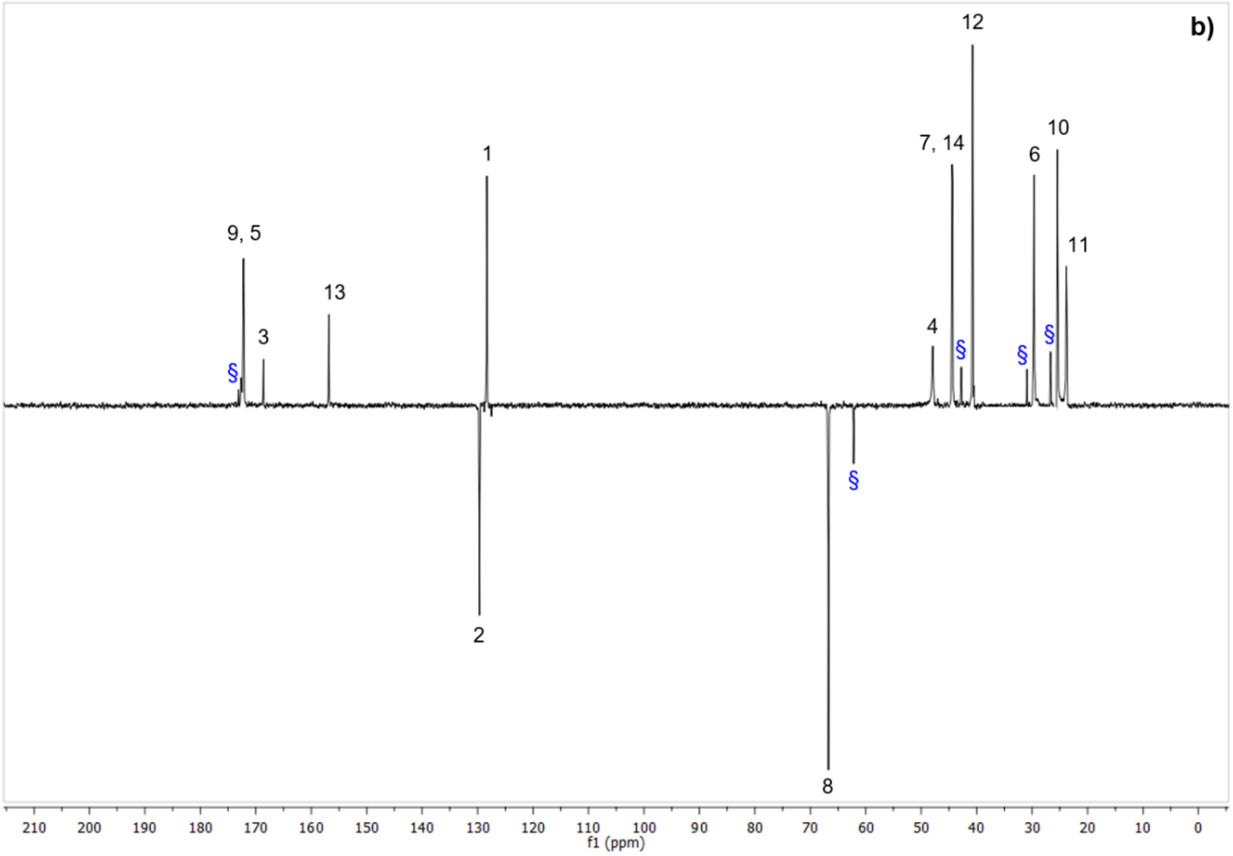

Figure S5. ^{1}H NMR (a) and ^{13}C APT NMR (b) spectra of MArMArMArMArM recorded in D_2O/DCl at pH = 4.5.

§ *L*-Arginine impurity

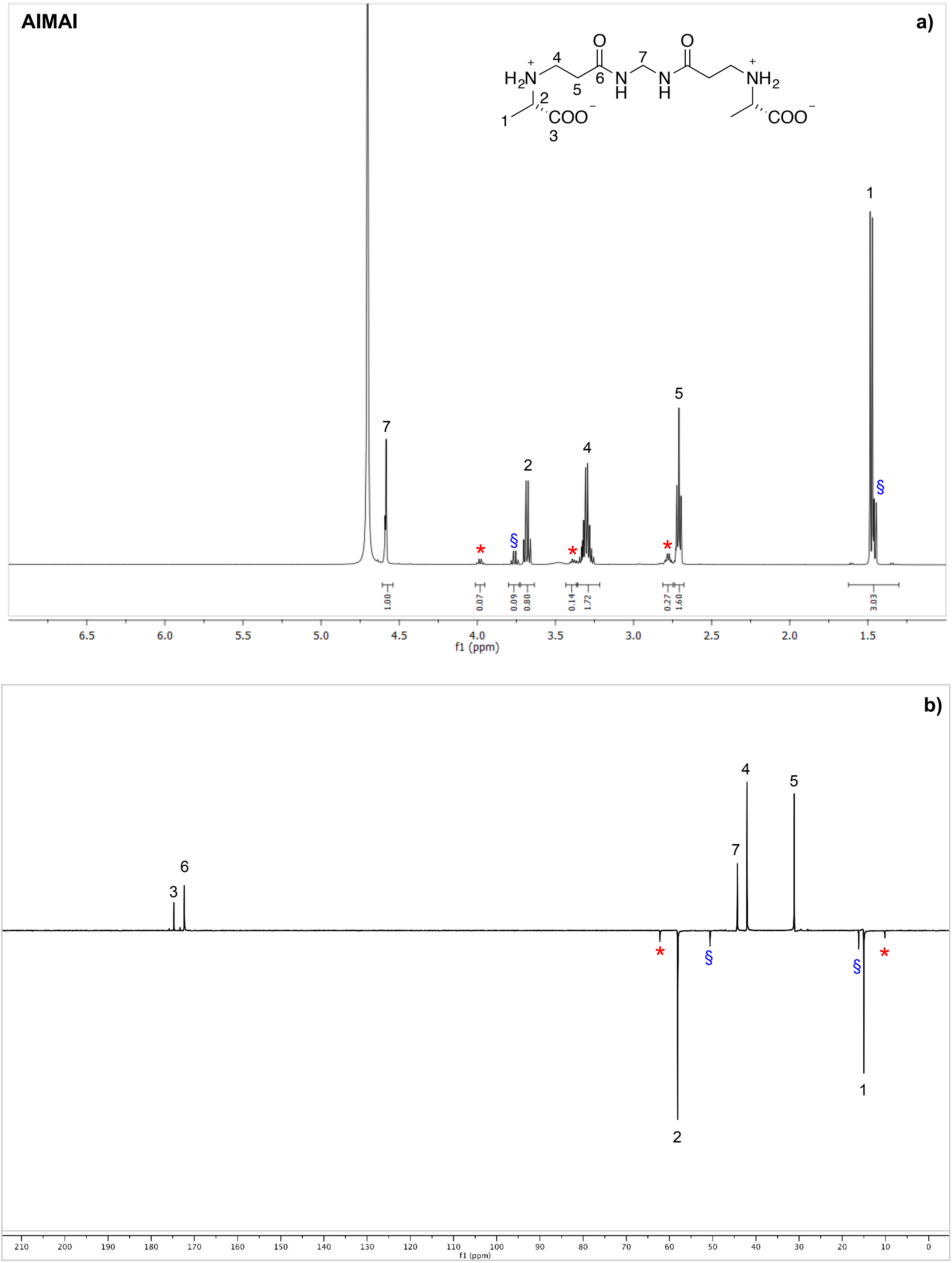

Figure S6. ^{1}H NMR (a) and ^{13}C APT NMR (b) spectra of AlMAl recorded in D_2O/DCl at pH = 4.5.

§ *L*-Alanine impurity

* Differently protonated species or other oligomers of the homologous series

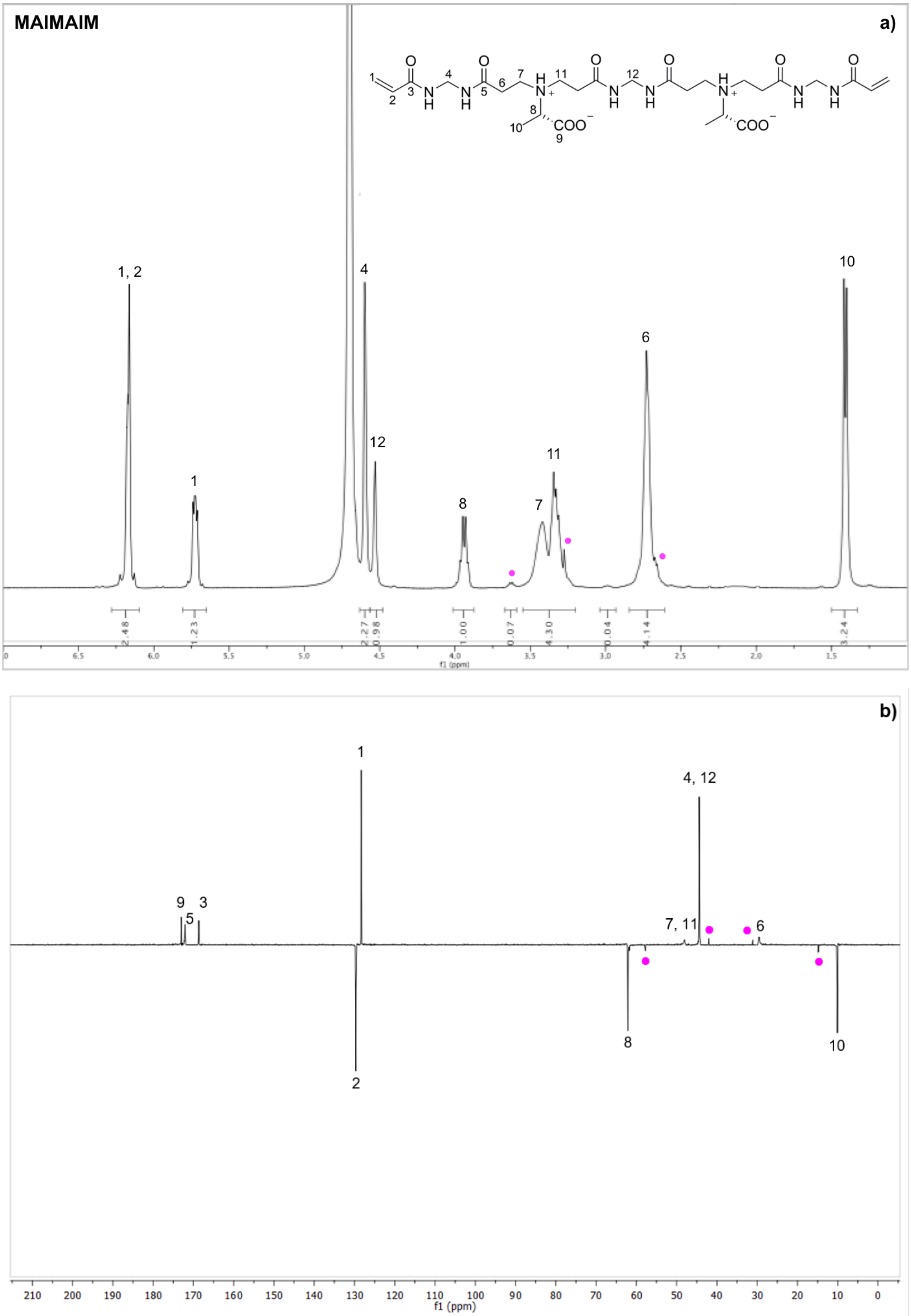

Figure S7. ^{1}H NMR (a) and ^{13}C APT NMR (b) spectra of MAlMAlM recorded in D_2O/DCl at pH = 4.5.

• Other oligomers of the homologous series

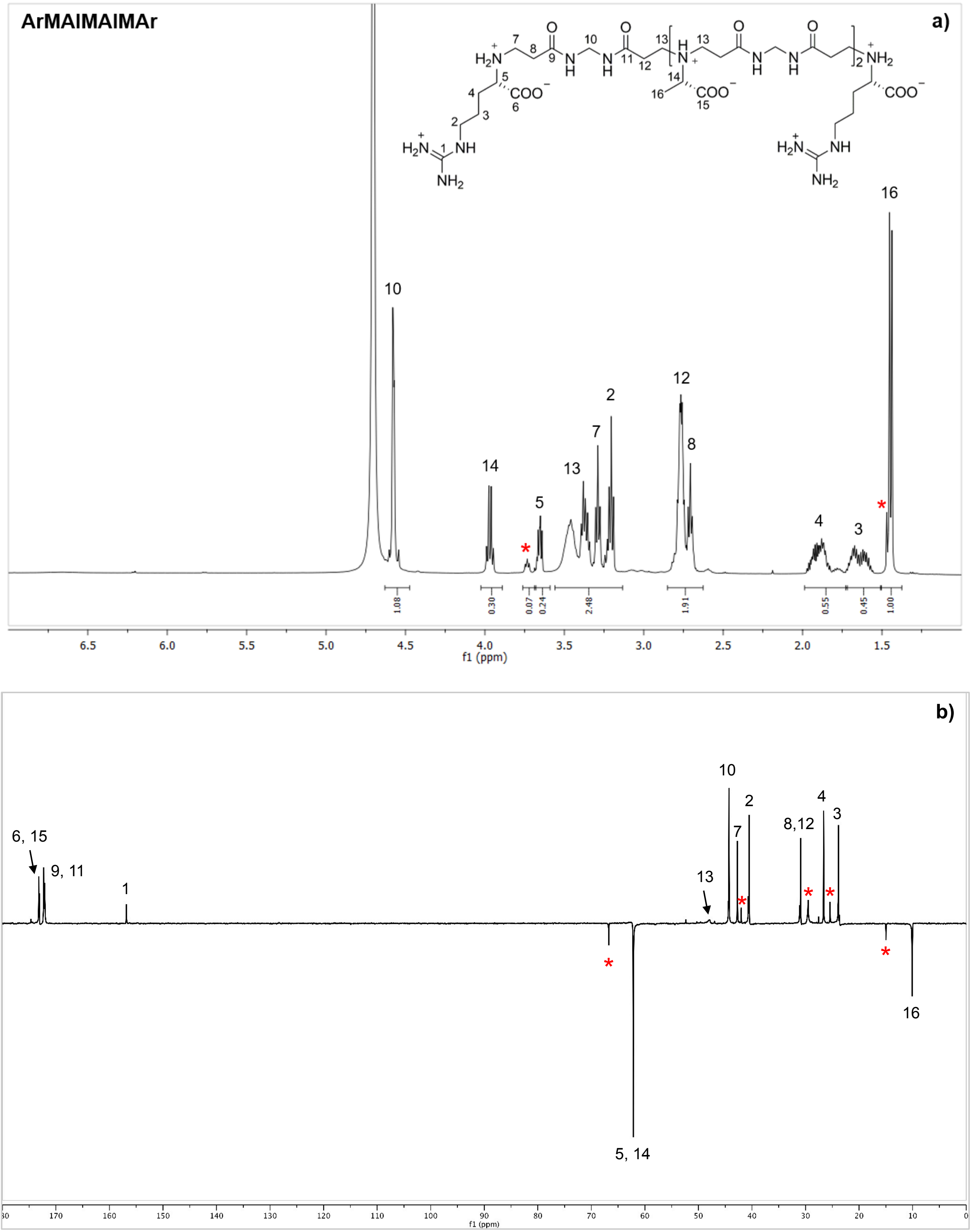

Figure S8. ^{1}H NMR (a) and ^{13}C APT NMR (b) spectra of ArMAlMAlMAr recorded in D_2O/DCl at pH = 4.5.

* Differently protonated species or other oligomers of the homologous series.

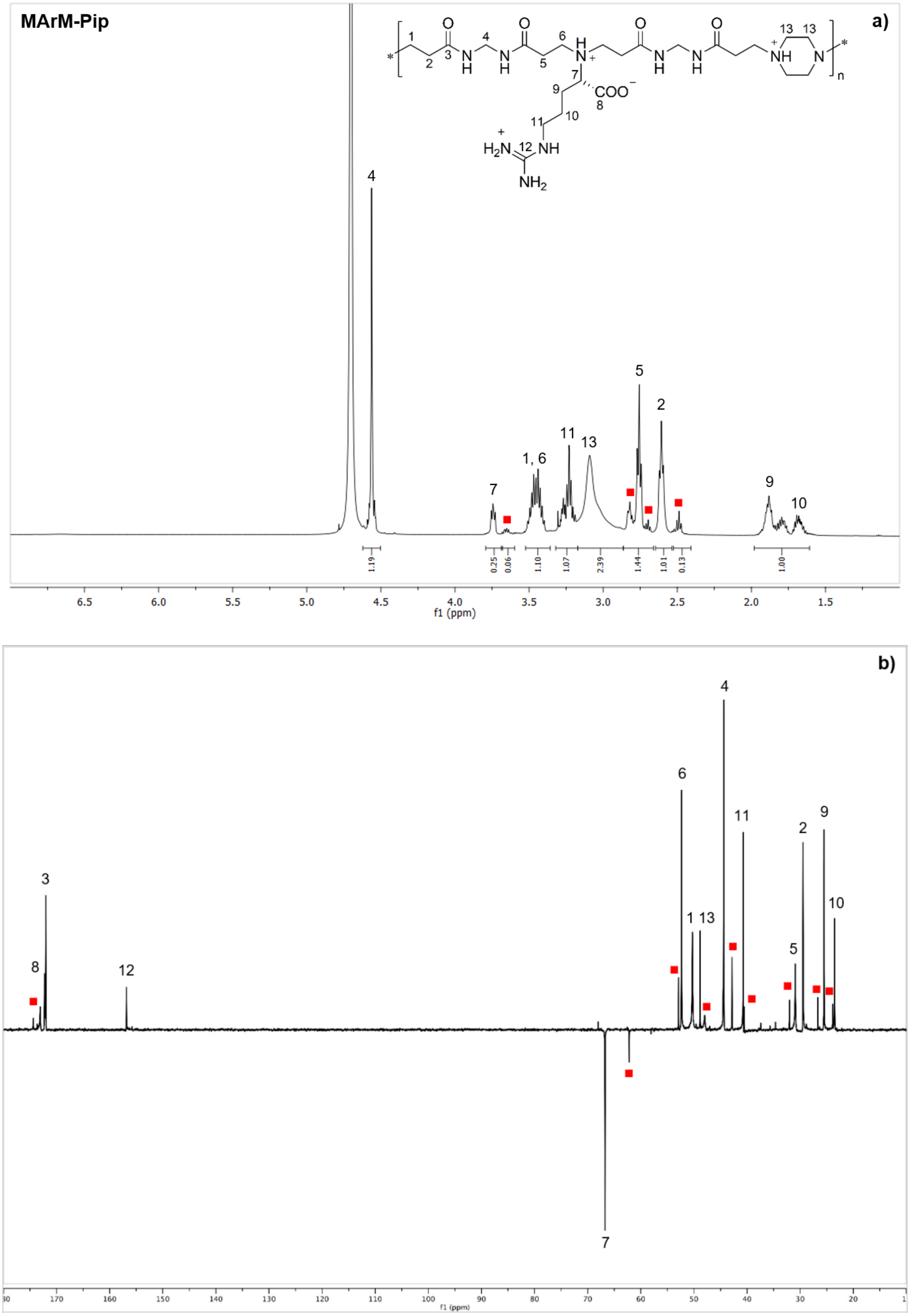

Figure S9. ^{1}H NMR (a) and ^{13}C APT NMR (b) spectra of [MArM-Pip]$_n$ recorded in D_2O/DCl at pH = 4.5.

■ Copolymer terminals

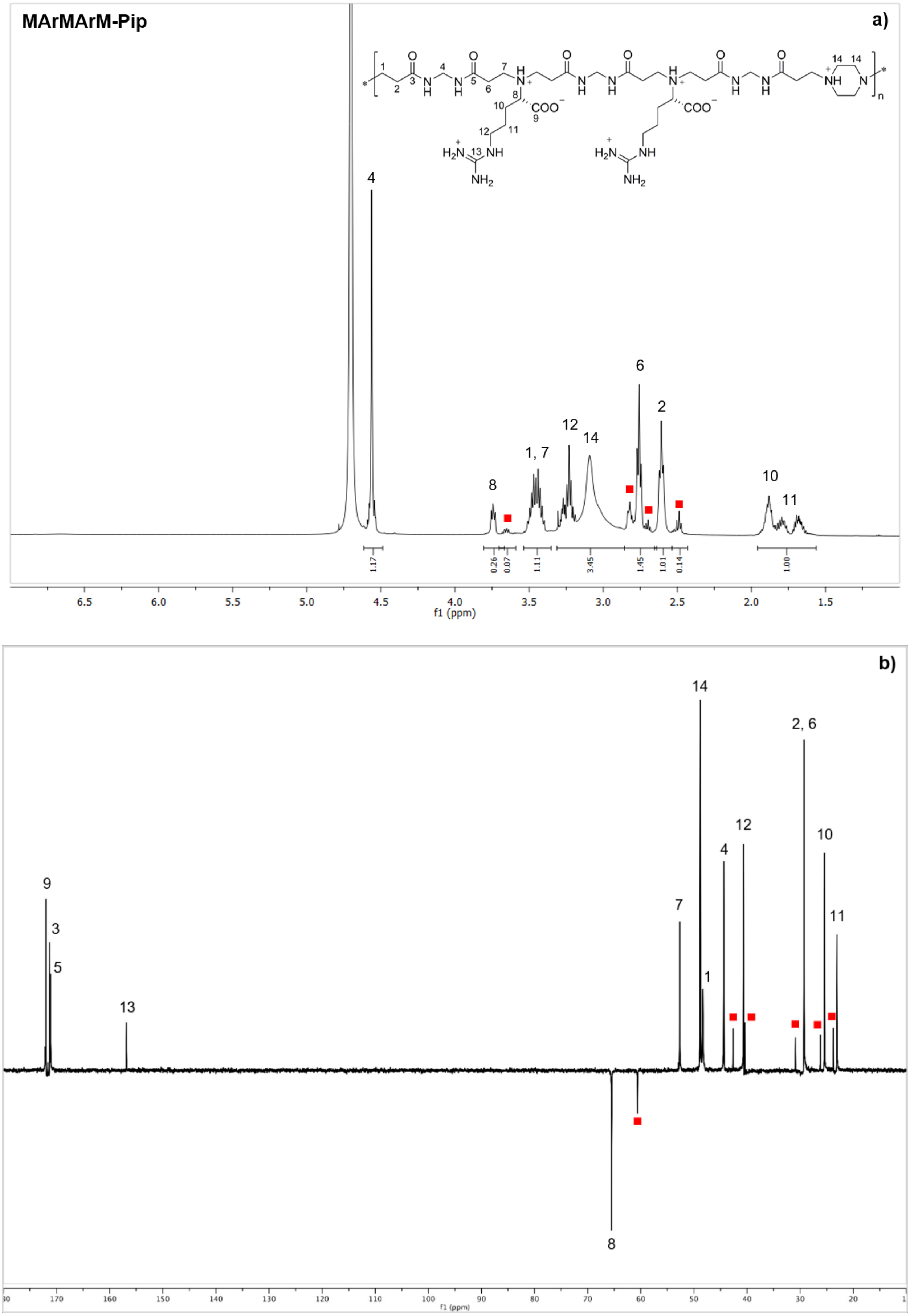

Figure S10. ^{1}H NMR (a) and ^{13}C APT NMR (b) spectra of $[MArMArM\text{-}Pip]_n$ recorded in D_2O/DCl at pH = 4.5.

■ Copolymer terminals

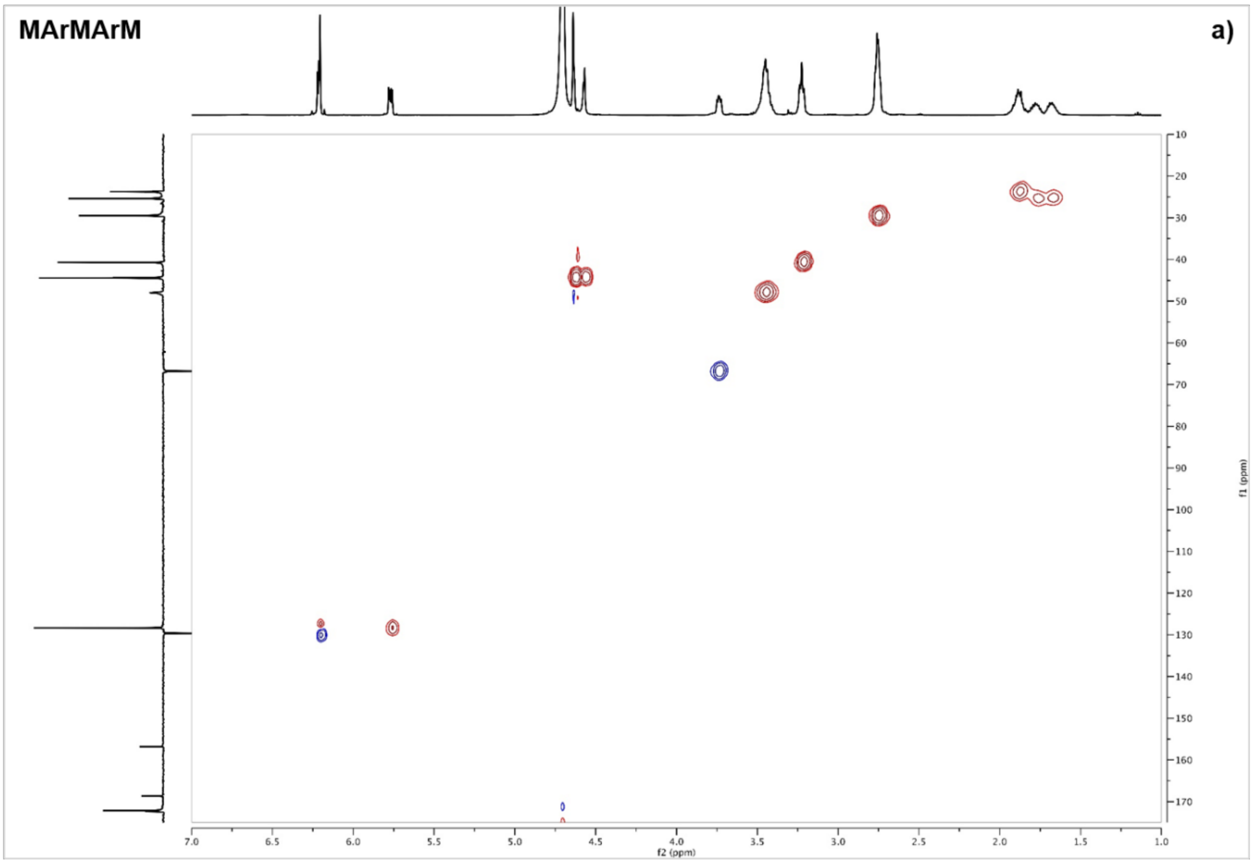
MArMArM
a)
f2 (ppm)
f1 (ppm)

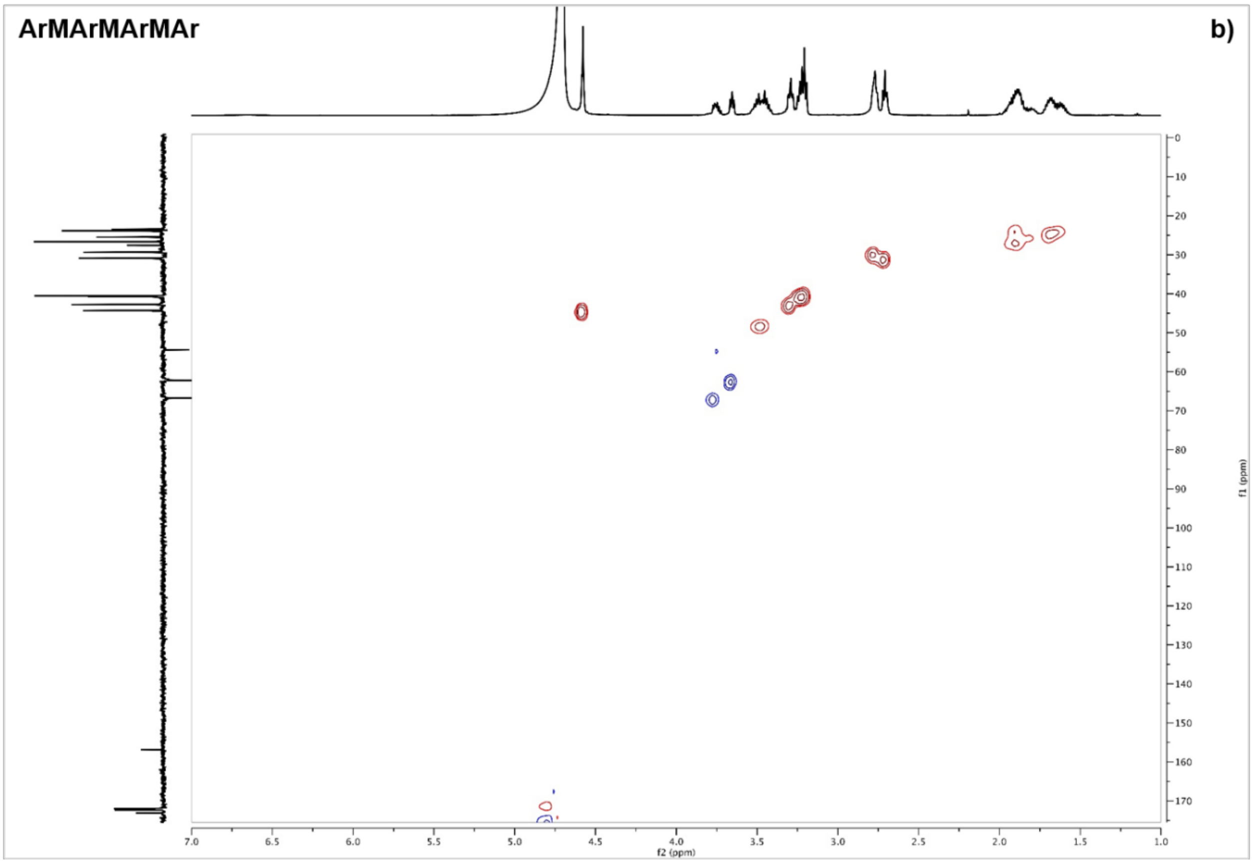
ArMArMArMAr
b)
f2 (ppm)
f1 (ppm)

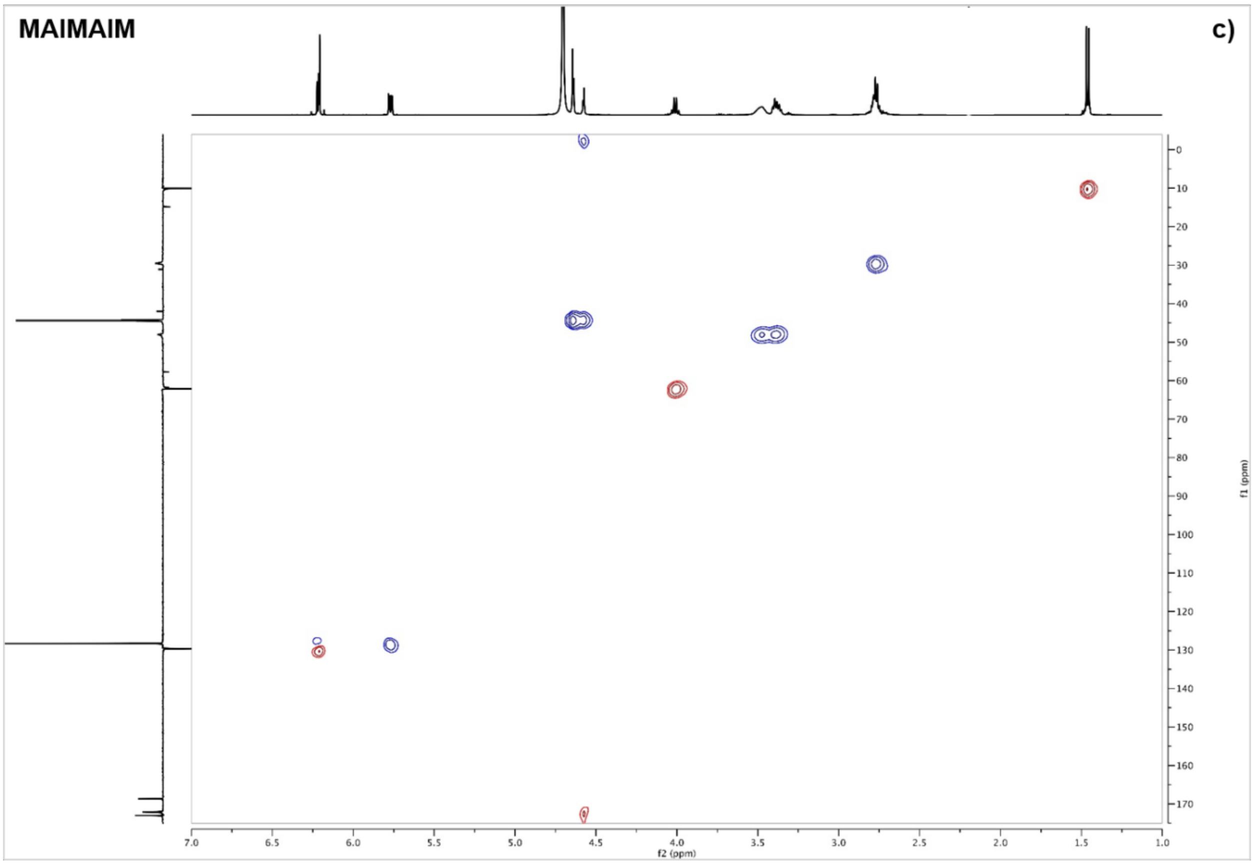

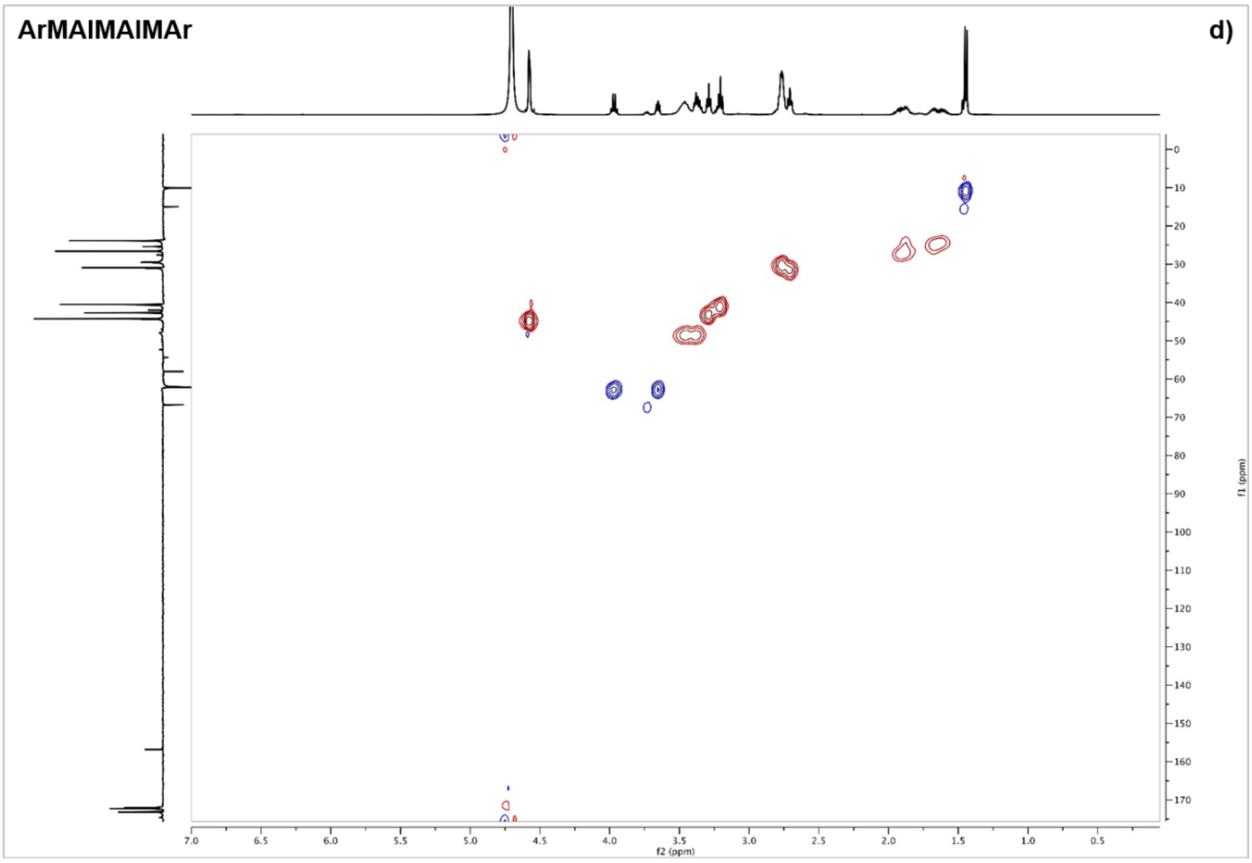

Figure S11. ^{1}H-^{13}C HSQC spectra of MArMArM (a); ArMArMArMAr (b); MAlMAlM (c); ArMAlMAlMAr (d) recorded in D_2O/DCl at pH = 4.5.

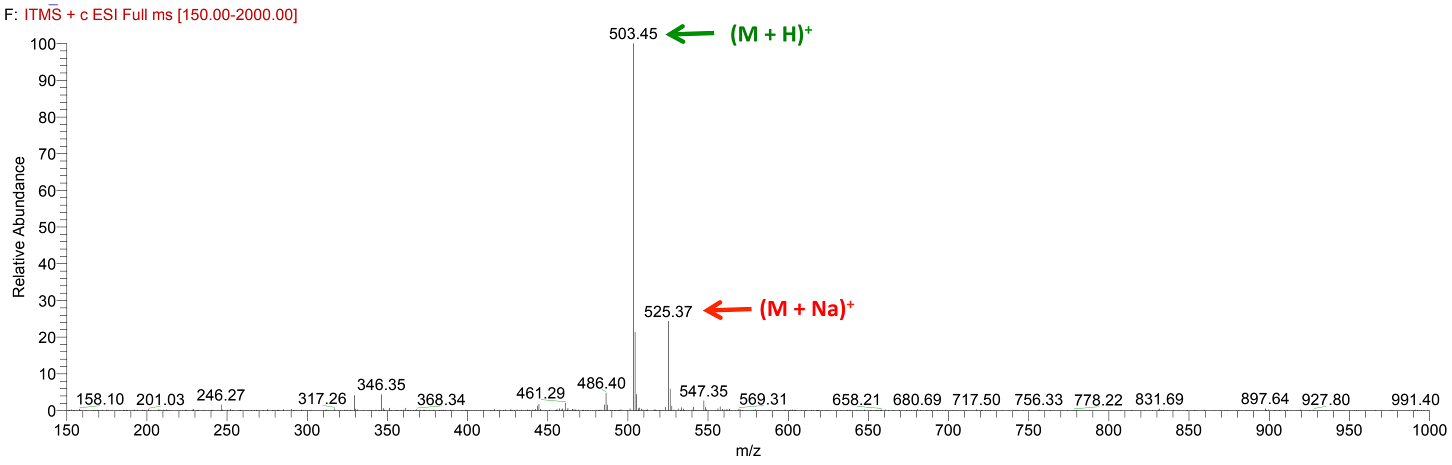

Figure S12. Positive ionization mode ESI-MS spectrum of ArMAr.

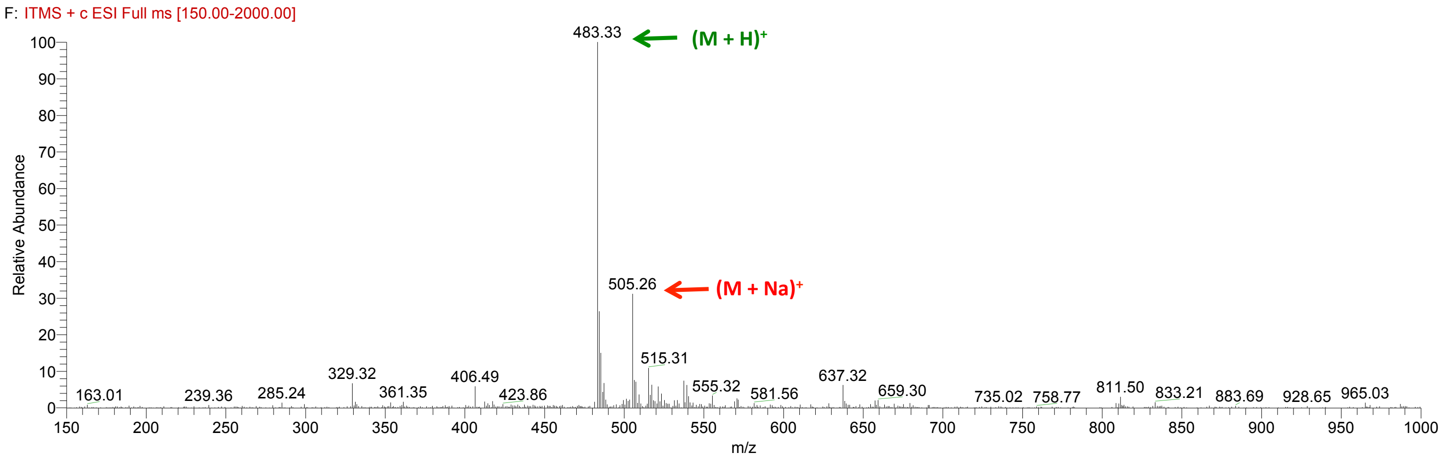

Figure S13. Positive ionization mode ESI-MS spectrum of MArM.

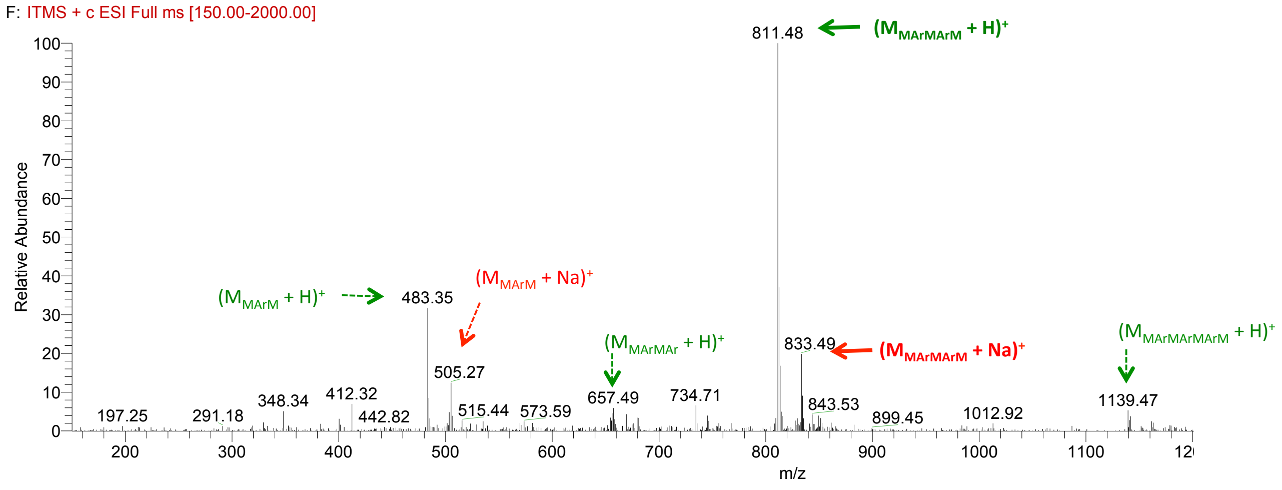

Figure S14. Positive ionization mode ESI-MS spectrum of MArMArM.

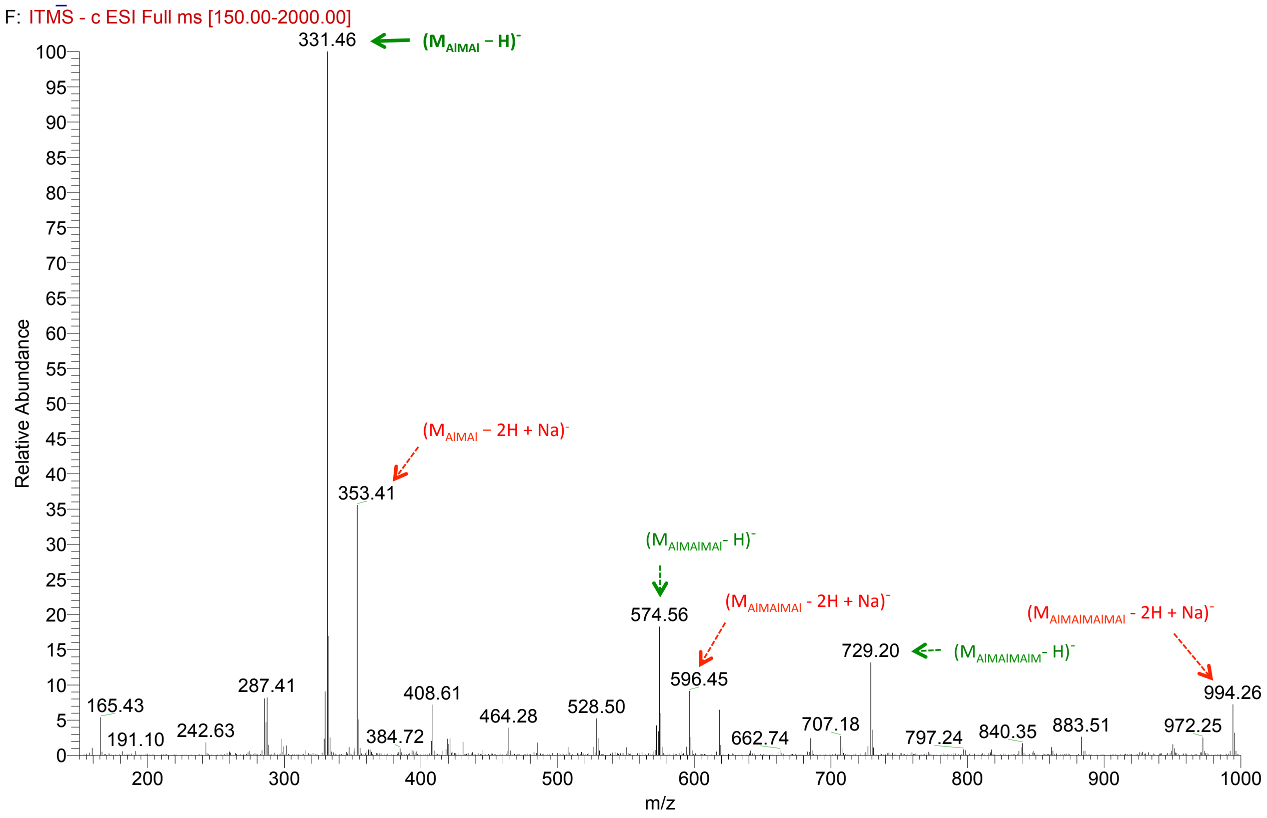

Figure S15. Negative ionization mode ESI-MS spectrum of AlMAl.

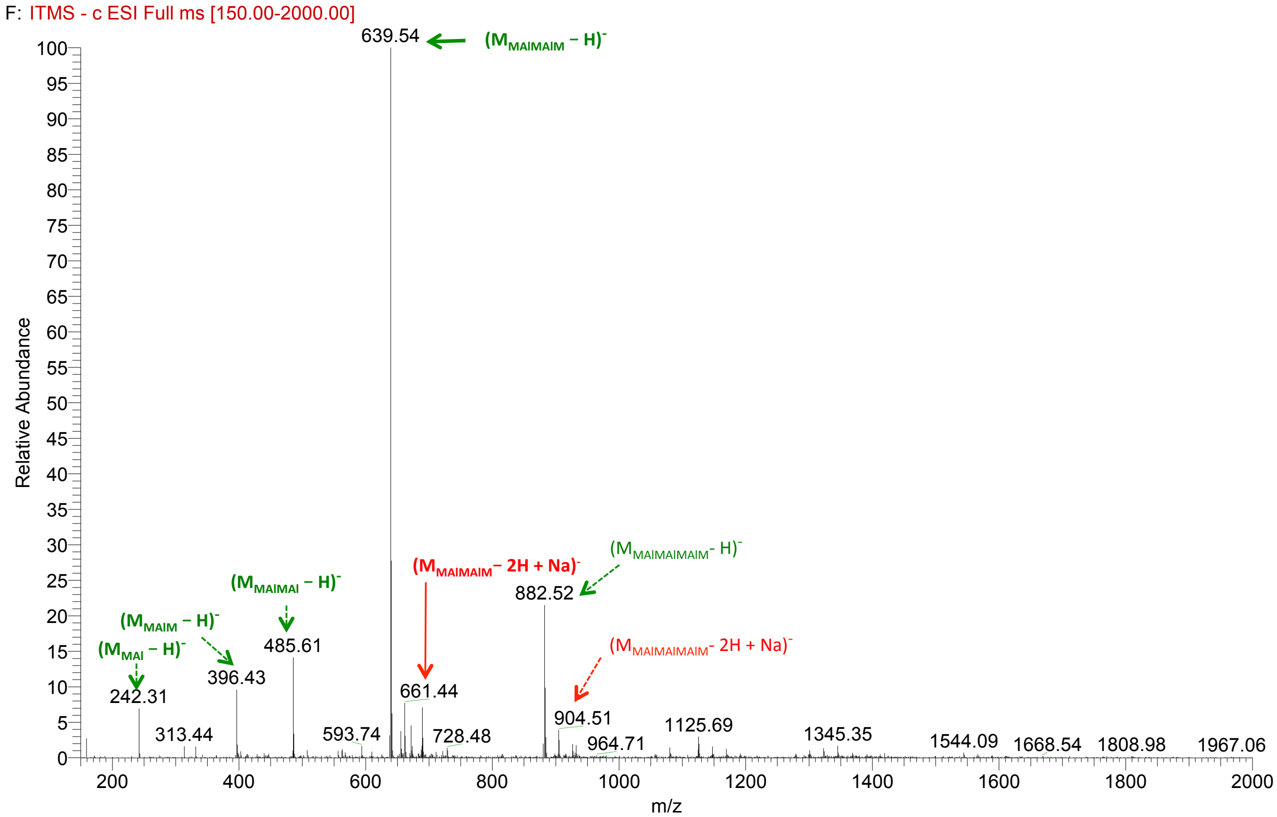

Figure S16. Negative ionization mode ESI-MS spectrum of MAlMAlM.

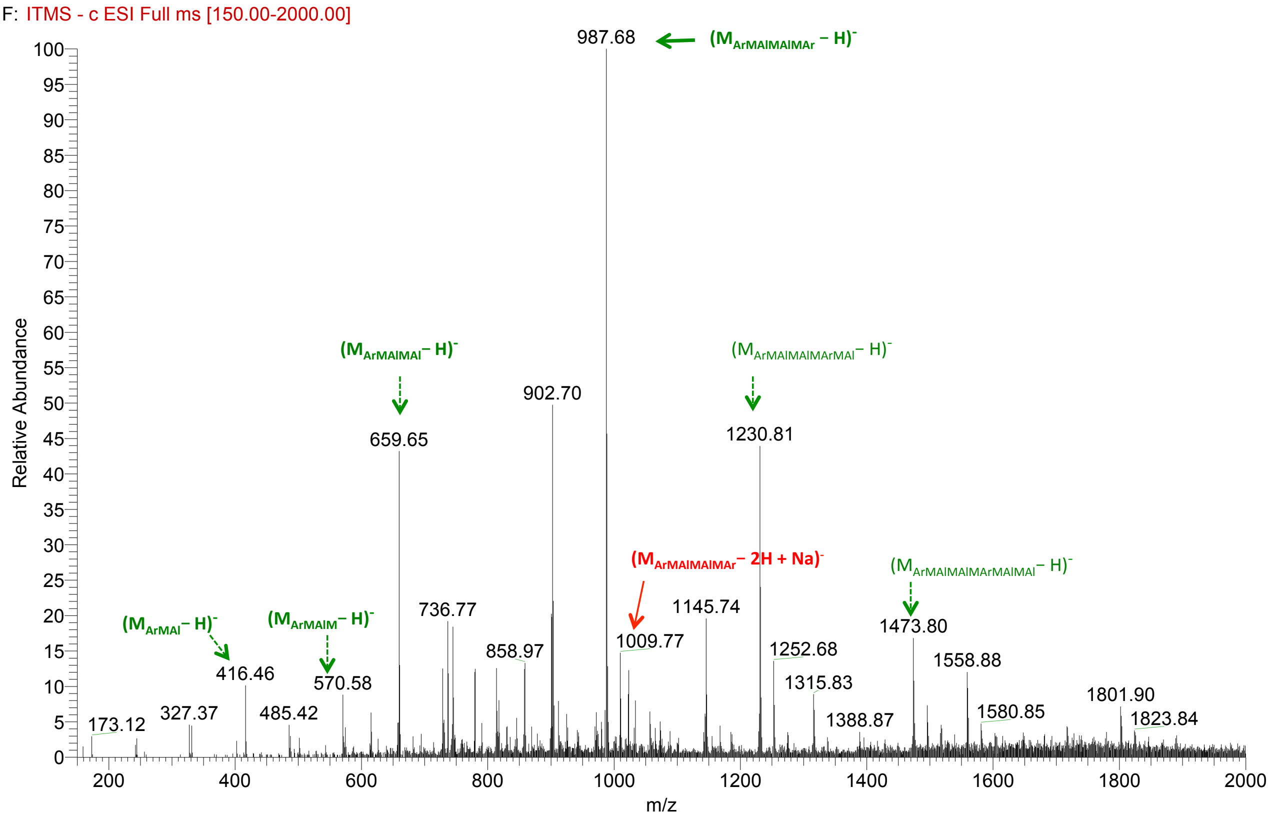

Figure S17. Negative ionization mode ESI-MS spectrum of ArMAlMAlMAr.

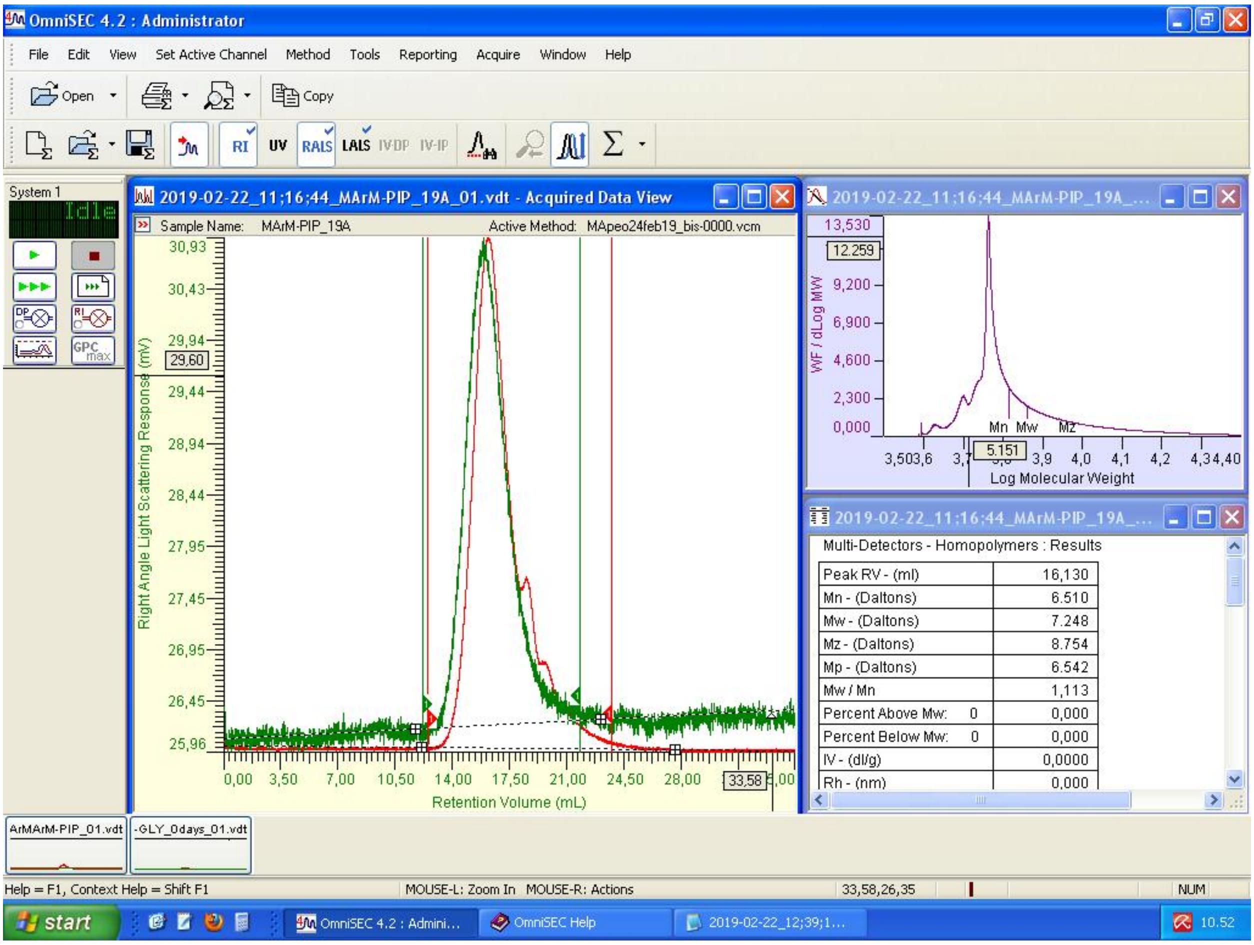

Figure S18. Snapshot of the OminiSEC 4.2 software output showing the results of SEC analysis of the $[MArM\text{-}Pip]_n$ copolymer carried out at pH 8.00±0.05 0.1 M Tris buffer solution with 0.2 M sodium chloride. The operational conditions were: sample concentration 20 mg mL^{-1}; flow rate 1 mL min^{-1}; injection volume 20 μL; column dimensions 300x7.5 mm^2, temperature 25 °C.

Green line: light scattering trace, red line: refractive index trace.

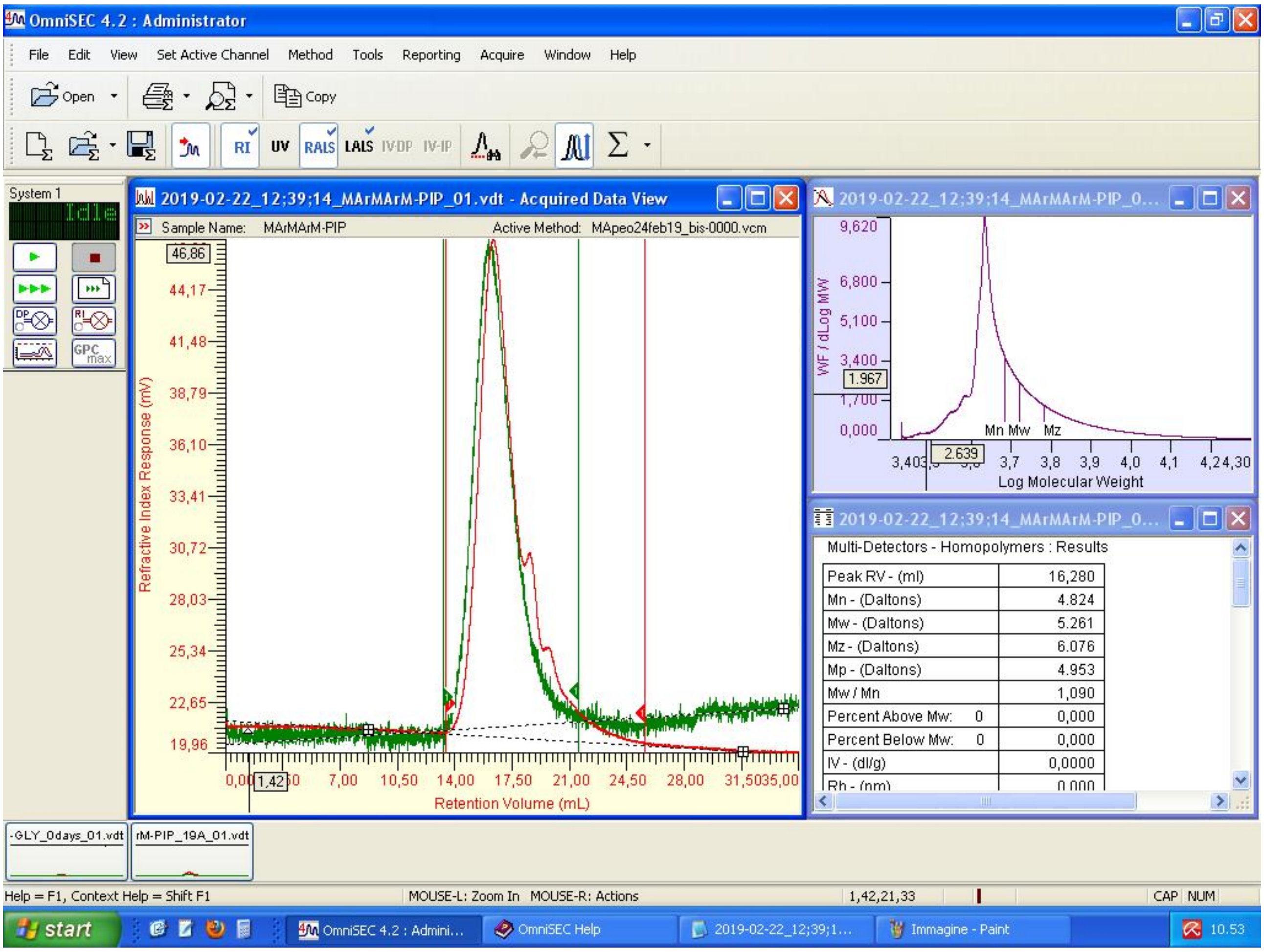

Figure S19. Snapshot of the OminiSEC 4.2 software output showing the results of SEC analysis of the [MArMArM-Pip]$_n$ copolymer carried out at pH 8.00±0.05 0.1 M Tris buffer solution with 0.2 M sodium chloride. The operational conditions were: sample concentration 20 mg mL^{-1}; flow rate 1 mL min^{-1}; injection volume 20 μL; column dimensions 300x7.5 mm^2, temperature 25 °C.

Green line: light scattering trace, red line: refractive index trace.
